# Human-centred mechanism design with Democratic AI

**DOI:** 10.1038/s41562-022-01383-x

**Published:** 2022-07-04

**Authors:** Raphael Koster, Jan Balaguer, Andrea Tacchetti, Ari Weinstein, Tina Zhu, Oliver Hauser, Duncan Williams, Lucy Campbell-Gillingham, Phoebe Thacker, Matthew Botvinick, Christopher Summerfield

**Affiliations:** 1 Deepmind, London, UK; 2 Department of Economics and Institute for Data Science and Artificial Intelligence, University of Exeter, Exeter, UK; 3 Gatsby Computational Neuroscience Unit, University College London, London, UK; 4 Department of Experimental Psychology, University of Oxford, Oxford, UK

**Keywords:** Science, technology and society, Economics

## Abstract

Building artificial intelligence (AI) that aligns with human values is an unsolved problem. Here we developed a human-in-the-loop research pipeline called Democratic AI, in which reinforcement learning is used to design a social mechanism that humans prefer by majority. A large group of humans played an online investment game that involved deciding whether to keep a monetary endowment or to share it with others for collective benefit. Shared revenue was returned to players under two different redistribution mechanisms, one designed by the AI and the other by humans. The AI discovered a mechanism that redressed initial wealth imbalance, sanctioned free riders and successfully won the majority vote. By optimizing for human preferences, Democratic AI offers a proof of concept for value-aligned policy innovation.

## Abstract (web summary)

Koster, Balaguer et al. show that an AI mechanism is able to learn to produce a redistribution policy which is preferred to alternatives by humans in an incentivized game.

## Main

The ultimate goal of AI research is to build technologies that benefit humans — from assisting us with quotidian tasks to addressing grand existential challenges facing society^1^. Machine learning systems have already solved major problems in biomedicine^2^, and helped address humanitarian and environmental challenges^3,4^. However, an underexplored frontier is the deployment of AI to help humans design fair and prosperous societies^5^. In economics and game theory, the field known as mechanism design studies how to optimally control the flow of wealth, information or power among incentivized actors to meet a desired objective, for example by regulating markets, setting taxes or aggregating electoral votes^6,7^. Here we asked whether a deep reinforcement learning (RL) agent could be used to design an economic mechanism that is measurably preferred by groups of incentivized humans.

The challenge of building AI systems whose behaviour is preferred by humans is called the problem of ‘value alignment’. One key hurdle for value alignment is that human society admits a plurality of views, making it unclear to whose preferences AI should align^8^. For example, political scientists and economists are often at loggerheads over which mechanisms will make our societies function most fairly or efficiently. In AI research, there is a growing realization that to build human-compatible systems, we need new research methods in which humans and agents interact^9–13^, and an increased effort to learn values directly from humans to build value-aligned AI^14^. Capitalizing on this idea, here we combined modern deep RL with an age-old technology for arbitrating among conflicting views—majoritarian democracy among human voters—to develop a human-centred research pipeline for value-aligned AI research. Instead of imbuing our agents with purportedly human values a priori, and thus potentially biasing systems towards the preferences of AI researchers, we train them to maximize a democratic objective: to design policies that humans prefer and thus will vote to implement in a majoritarian election. We call our approach, which extends recent related participatory approaches^11,14,15^, ‘Democratic AI’.

As a first rigorous test, we deploy Democratic AI to address a question that has defined the major axes of political agreement and division in modern times: when people act collectively to generate wealth, how should the proceeds be distributed?^16–21^. We asked a large group of humans to play an incentive-compatible online investment game that involved repeated decisions about whether to keep a monetary endowment or to share it with other players for potential collective benefit. We trained a deep RL agent to design a redistribution mechanism which shared funds back to players under both wealth equality and inequality. The mechanism it produced was ultimately preferred by the players in a majoritarian election.

## Results

**Fig. 1:**
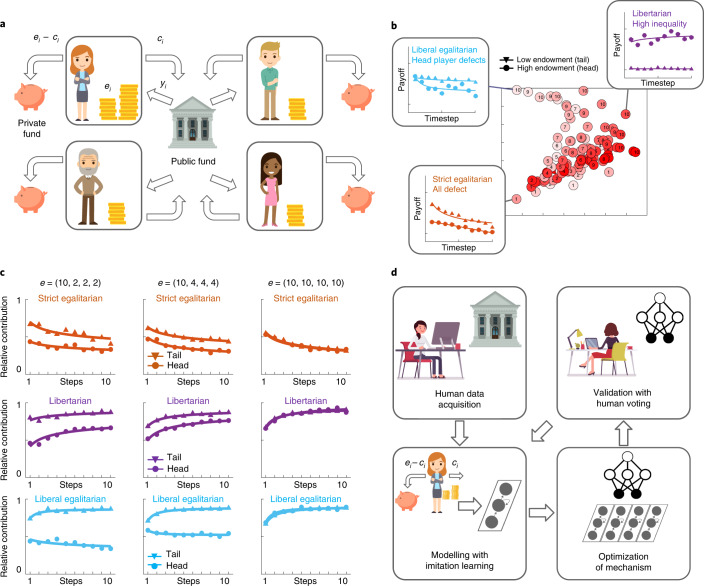
Illustration of the game and Experiment 1. **a**, Illustration of the setup of the investment game. **b**, The ideological manifold for endowment distribution (10, 2, 2, 2). The plot shows a visualization of a space of redistribution mechanisms defined by parameters *w* and *v* in two dimensions. Each red dot is a mechanism, and distances between dots conserve dissimilarities in the (average) relative payout to virtual players (both head and tail). Dot numbers denote bins of mechanism parameter *w* (1, lowest; 10, highest) and shading denotes bins of *v* (light, more relative; dark, more absolute). Inset, example payouts to head (circles) and tail (triangles) players under the canonical mechanisms used as baselines against which to test the AI. Under strict egalitarian, payouts decline to head and tail players. Under libertarian, there is great inequality between head and tail players. Under liberal egalitarian, the head player stops contributing, so payouts decline for both head and tail players. **c**, Average relative contributions (as a fraction of endowment) over 10 rounds (*x* axis) in Exp. 1 for three different initial endowment conditions. Under strict egalitarian redistribution, tail player (triangles) contributions are higher when initial endowments are lower, but head player (circles) contributions do not differ. Under libertarian, head player contributions increase with equality, but tail player contributions remain constant. Head player contributions increase strongly with endowment under liberal egalitarian. **d**, Illustration of our agent design pipeline.

We tested Democratic AI using a mechanism design problem based on an economic game. The game generalizes the linear public goods problem that has been extensively used to study human collective action^22,23^ (Fig. 1a). In each of 10 rounds, each player *i* contributes an integer number *c*_i_ of coins to a public investment fund, drawing upon an endowment *e*_i_, with the residual sum *e*_*i*_−*c*_*i*_ remaining in a private account (endowments may vary across players, with one player receiving more than the others). Aggregated contributions over *k* = 4 players are scaled by a growth factor *r* = 1.6 (positive return on investment; this is equivalent to a marginal per capita return (MPCR) of 0.4). The public fund is paid back to players under a redistribution mechanism which specifies the fraction of total public investment that is returned to each player, conditional on their contribution and endowment. This game admits a continuum of mechanisms for redistribution popularly associated with opposing ends of the political spectrum^19^, in which returns variously depend on the contributions of self and others^23^.

### Experiment 1

We illustrate the richness of the mechanism design problem in Exp. 1, in which we measured human contributions made under three canonical redistribution principles: strict egalitarian, libertarian and liberal egalitarian. Players (*n* = 756) were assigned to groups of 4 players, with one head player who received 10 coins endowment and three tail players who received either 2, 4 or 10 coins (head/tail labels were nominal in the latter condition). Thus, endowments were unequal when tail players received less than 10 coins and equal when all players received 10 coins. Each group played multiple games each of 10 rounds, receiving the same endowment on each occasion, but experiencing each game under a different redistribution mechanism (see Supplementary Methods). Each redistribution mechanism determined the payout *y*_*i*_ received by player *i* as a different function of the public contribution of both self and others.

The strict egalitarian redistribution mechanism divides public funds equally among all players irrespective of their contributions^24^. It thus recreates the linear public goods game that, for *r* < k, is a social dilemma in that each individual benefits from withholding contributions and free riding on the largesse of other players^23^. Accordingly, contributions under this mechanism decline over time (effect of time on contributions for head player: 2 coins, *F*_9,540_ = 2.51, *P* = 0.023, *η*^2^ = 0.017; 4 coins, *F*_9,540_ = 5.5, *P* < 0.001, *η*^2^ = 0.041; 10 coins, *F*_9,594_ = 7.27, *P* < 0.001, *η*^2^ = 0.056; Fig. 1c, top), mirroring previously described results^22,23^. The libertarian mechanism^21^ returns a payout to each player in proportion to their contribution ${{{y}}}_{{{i}}} = {{{r}}} \times {{{c}}}_{{{i}}}$ such that *c*_*i*_ = *e*_*i*_ is a pareto-efficient Nash equilibrium. This mechanism effectively privatizes contributions and removes the social dilemma, encouraging players to increase their contributions (effect of time on contributions for head player: 2 coins, *F*_9,297_ = 9.96, *P* < 0.001, *η*^2^ = 0.062; 4 coins, *F*_9,234_ = 9.55, *P* < 0.001, *η*^2^ = 0.073; 10 coins, *F*_9,270_ = 12.56, *P* < 0.001, *η*^2^ = 0.013; Fig. 1c, middle) as observed previously^25^ (note that while players receive detailed instructions about the game dynamics, they are obliged to learn about each mechanism from experience). Finally, liberal egalitarianism proposes that each player is accountable for their actions but not initial advantage, and so payout depends on the fraction of endowment that is contributed^26^. When payouts were relative to endowment-normalized contributions (liberal egalitarian), the tail players learned rapidly to contribute (2 coins, *F*_9,720_ = 4.79, *P* < 0.001, *η*^2^ = 0.025; 4 coins, *F*_9,909_ = 15.74, *P* < 0.001, *η*^2^ = 0.043) but the head player’s contributions remained flat (2 coins, *F*_9,234_ = 1.84, *P* = 0.139, *η*^2^ = 0.017; 4 coins, *F*_9,297_ = 0.62, *P* = 0.601, *η*^2^ = 0.004), diminishing the availability of public funds.

Previous reports have suggested that heterogeneity of endowment or MPCR can influence contribution to the public fund^27,28^, especially when inequality is made salient to participants^29^. Here we observed a comparable phenomenon when we examined the contributions of the head player (who received 10 coins) as a function of the endowment received by tail players, which could be either equal or lower. Under strict egalitarian, the head player contributed the same irrespective of the endowment of others (*F*_2,188_ = 0.29, *P* = 0.745, *η*^2^ = 0.003), but under liberal egalitarian the head player was less prone to contribute when others were less well off (*F*_2,377_ = 14.10, *P* < 0.001, *η*^2^ = 0.070; the effect for libertarian was not significant: *F*_2,280_ = 2.82, *P* = 0.069, *η*^2^ = 0.020). Thus, productivity was dampened under conditions of greater inequality.

More generally, Exp. 1 highlights the challenge that the game poses for the mechanism designer: a redistribution scheme might be unpopular because it provokes a general collapse of contributions due to free riding, leads to unequal outcomes, or siphons funds away too aggressively from the wealthiest player, who then fails to provision the public fund. We thus asked whether an AI system could design a mechanism that humans preferred over these alternatives.

### Human-in-the-loop pipeline

How then should the public funds be shared? The maximally popular policy could be one of these three canonical mechanisms or something else entirely. The size of the potential search space makes it hard to identify the preferred mechanism using traditional behavioural research methods. We thus developed a human-in-the-loop AI research pipeline to tackle this problem (Fig. 1d). First, we collected an initial sample of human data (Acquire) and used it to train ‘virtual human players’ which were recurrent neural networks that learned to imitate human behaviour during the game and voted according to the same principles as human players (Model; see Supplementary Fig. 1). This simulation step was necessary because training agents during online interaction with humans would have been prohibitively costly and time-consuming. Third, we optimized the mechanism design with deep RL, using policy gradient methods^30^ to maximize the votes of virtual human players (Optimize). Fourth, we sampled a new group of humans, and pitted the RL-designed redistribution mechanisms against rival baselines in a series of head-to-head majoritarian elections. This new human data was then used to augment our player modelling process, which in turn improved optimization and led to potentially better mechanisms (Repeat). This pipeline builds on recent approaches that have used human data interactively to train artificial agents^31–33^. We iterated this procedure to obtain a mechanism that we call the Human Centred Redistribution Mechanism or HCRM, which is the major focus of the remainder of this report.

### The ideological manifold

Before evaluating HCRM with a new group of human players, we used our research pipeline to determine which baseline mechanisms might pose the strongest competition. To achieve this, we generalized the three canonical baselines to produce a continuously parameterized space of redistribution mechanisms. We first assume that the fractional payout to each player *y*_*i*_ is composed of an absolute $({{{y}}}_{{{i}}}^{{{{\mathrm{abs}}}}})$ and relative (${{{y}}}_{{{i}}}^{{{{\mathrm{rel}}}}}$) component, which are combined via a mixing parameter *v*. These components are in turn given by contributions from both the focal and other players (mixing parameter *w*; see below and Methods). In these baselines, the payout to player *i* is thus given by

$${{{y}}}_{{{i}}} = {{{v}}}\left( {{{{y}}}_{{{i}}}^{{{{\mathrm{rel}}}}}} \right) + (1 - {{{v}}})({{{y}}}_{{{i}}}^{{{{\mathrm{abs}}}}}),$$

where the absolute component combines their own contribution *c*_*i*_ with the average of that from other players *c*_*−i*_ so that

$${{{y}}}_{{{i}}}^{{{{\mathrm{abs}}}}} = {{{r}}}[{{{w}}}\left( {{{{c}}}_{{{i}}}} \right) + (1 - {{{w}}})({{{c}}}_{ - {{{i}}}})]$$

and the relative component is similarly determined by ${\uprho}_{{{i}}} = {{{c}}}_{{{i}}}/{{{e}}}_{{{i}}}$, which is the ratio of contribution to endowment for player *i*:

$${{{y}}}_{{{i}}}^{{{{\mathrm{rel}}}}} = {{{r}}}\left( {\frac{{{{C}}}}{{{{P}}}}} \right)[{{{w}}}({\uprho}_{{{i}}}) + \left( {1 - {{{w}}}} \right)\left( {{\uprho }}_{ - {{{i}}}} \right)],$$

where *c*_−*i*_ and *ρ*_−*i*_ are respectively the average contributions and ratios from players other than *i*, and *C* and *P* are the sum of contributions and ratios across all players.

We call this space of baseline mechanisms defined by *v* and *w* the ideological manifold (Fig. 1b). We note that the three baseline mechanisms we have considered so far lie within this space: libertarian (${{{w}}} = 1,{{{v}}} = 0$), liberal egalitarian (${{{w}}} = 1,{{{v}}} = 1$) and strict egalitarian (${{{w}}} = 1/{{{k}}}$). We explore the properties of these mechanisms in more detail in Supplementary Fig. 10.

We sampled mechanisms from the ideological manifold and pitted them against one another exhaustively in a two-player tournament. Our goal was to identify the mechanism that maximized votes among the virtual human players (neural networks that had been trained to imitate real human behaviour). This exercise identified liberal egalitarian as the single Nash equilibrium of the two-player tournament and thus not only as the strongest competitor among the three canonical baselines but also among the entire space of mechanisms (Supplementary Table 2).

### Experiment 2

**Fig. 2:**
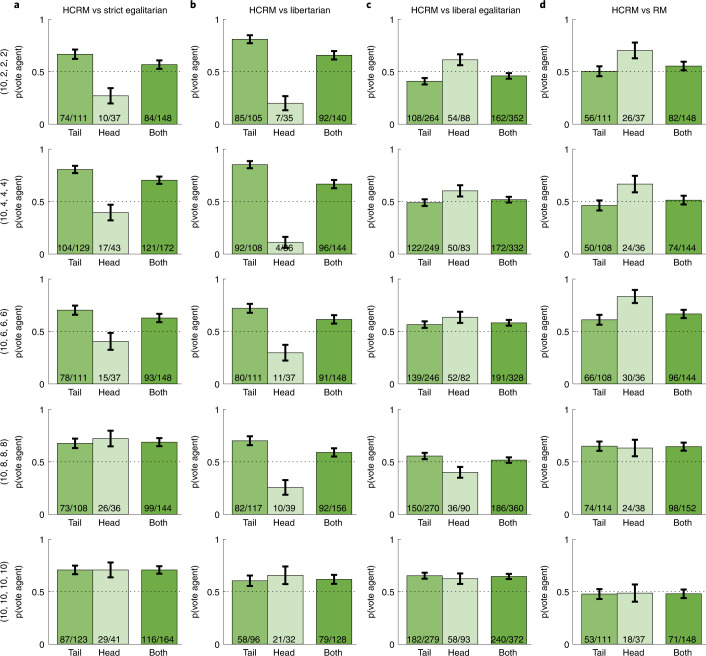
Overall vote share by endowment and rival mechanism. **a**–**d**, Vote share for the HCRM against the three canonical baselines (**a**–**c**) and the RM (**d**) for each endowment condition. The three bars show the average number of votes for the agent given by the tail players, the head player and all players. In all plots, bars show binomial standard error.

Armed with this intuition, in Exp. 2a–c we evaluated the AI-designed HCRM against the three canonical baselines introduced above. Groups of 4 human participants (${{{n}}} = 2,508$) played successive incentive-compatible games of 10 rounds under two rival mechanisms, before voting for one that they preferred to play again (for additional payoff) in a final round. We randomized players into five endowment conditions, in which a head player received ${{{e}}}_{{{{\mathrm{head}}}}} =$10 coins endowment and three tail players received the same ${{{e}}}_{{{{\mathrm{tail}}}}} \in \left\{ {2,4,6,8,10} \right\}$ (once assigned, endowments remained constant throughout the game). We found that HCRM was more popular than all three baselines (Fig. 2a–c and Supplementary Table 1a; all *P* values obtained below are obtained from one-tailed binomial tests corrected for correlated responses within the group; see Methods), obtaining a total of 513/776 (66.2%) votes against strict egalitarian (*P* < 0.001), 450/740 (60.8%) against libertarian (*P* < 0.001) and 951/1,744 (54.5%) against liberal egalitarian (*P* < 0.001).

Against strict egalitarian and libertarian, the AI-designed mechanism was also more popular under all five endowment distributions tested, ranging from full equality to the most unequal endowment condition ((10, 2, 2, 2) implies a Gini coefficient (a measure of wealth inequality) of 0.38, roughly equivalent to contemporary Russia). Across these conditions, its vote share ranged from 56.0% to 67.0% against egalitarian and from 57.5% to 66.7% against libertarian. Consistent with the data from our two-player tournament, liberal egalitarian proved popular among humans and thus more difficult to beat. Indeed, although HCRM was preferred overall, under full equality (64.5%, *P* < 0.001) and under moderate inequality (endowments (10, 8, 8, 8) and (10, 6, 6, 6)) with a vote share of 54.5% (*P* < 0.006), there was no reliable difference in voting preference between HCRM and liberal egalitarian under the most unequal conditions (HCRM vote share 47.4%, *P* = 0.897), suggesting that liberal egalitarian redistribution offered an equally good alternative to HCRM under conditions of highest inequality.

Our AI-designed HCRM was trained by interacting with neural networks that imitated human behaviour. However, if our participants are rational agents who learn to maximize their return over the course of each game, then it should be possible to solve the problem without recourse to human training data at all, by substituting our virtual human players for a new class of rational players that are trained to maximize their own expected return within the game (see Methods). Previous work has implied that successful human-centred mechanisms can be obtained in this fully multi-agent setting^34,35^. Alternatively, if modelling human cognitive biases is critical, then a system trained to maximize the votes of rational players may transfer more poorly back to human participants. In Exp. 3, we tested this by exposing a new group of human participants (*n* = 736) to both the mechanism designed by HCRM and that proposed by a new rational mechanism (RM) that was trained with rational players but otherwise identical.

### Experiment 3

Overall, 57.2% (421/736) of participants preferred HCRM over the RM (*P* < 0.001; Fig. 2d). Interestingly, RM learned a radical policy under unequal endowments that neglected the head player and paid out principally to the tail players (Supplementary Fig. 3). Despite the favourable ratio of tail to head players, however, this was unsuccessful even in the most unequal endowment conditions, because the head player rapidly stopped contributing to the detriment of everyone (including the tail players), leading to an overall lower group surplus than for HCRM (*t*_183_ = 7.96, *P* < 0.001). In other words, the redistribution policy that humans prefer is neither one that shares out public funds equally, nor one that tries to speak only to the interests of a majority of less well-endowed players. One exception was under equal endowment, where HCRM and RM performed nearly identically (HCRM vote share 71/148 or 47.9%, *P* = 0.617), implying that for the setting we explored, rational models may offer a good account of human behaviour when the initial conditions are fair. Together, however, these results imply that human data may be crucial when using AI for mechanism design.

**Fig. 3:**
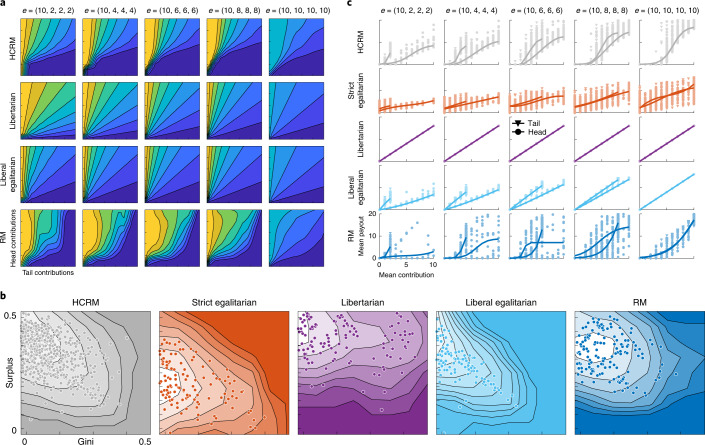
Analysis of HCRM mechanism. **a**, ‘Beach plots’ were created by simulating virtual players. They show revenue share allocated to head player as a function of contribution (relative to endowment) of head vs tail player. Warmer (colder) colours indicate relatively more funds redistributed to head (tail) player. **b**, Two-dimensional distributions of surplus (in log units) and Gini coefficient for each group under each mechanism. Lighter colours indicate higher density. Higher surplus implies greater productivity; lower Gini implies greater equality. Each dot is a game. **c**, Empirically observed relationship between contributions and payouts for each mechanism and endowment condition. Each dot is a head player or the average of tail players in a single game. Shading shows the density of dots. Lines are fit separately to head and tail players.

The RL mechanism designer was not equipped with memory, hence HCRM is readily interpretable, that is, it can be transparently described as a two-dimensional surface that maps the relative contribution of head and tail players to their share of the proceeds (‘Beach plots’ in Fig. 3a). This allowed us to ask why the RL-designed mechanism is popular with human players. RL discovered a hybrid mechanism that eschewed traditionally proposed redistribution schemes that emphasize individual discretion over resource allocation (libertarian) or collective equality (strict egalitarian). Pursuing a broadly liberal egalitarian policy, HRCM sought to reduce pre-existing income disparities by compensating players in proportion to their contribution relative to endowment. In other words, rather than simply maximizing efficiency, the mechanism was progressive: it promoted enfranchisement of those who began the game at a wealth disadvantage, at the expense of those with higher initial endowment. In doing so, it achieved a favourable trade-off between productivity (surplus) and equality (Gini coefficient) among rival mechanisms (Fig. 3b; see also Supplementary Fig. 3). However, unlike liberal egalitarian, it returned almost nothing to players unless they contribute approximately half their endowment (Fig. 3c). In other words, RL effectively discovers that humans facing social dilemmas prefer mechanisms that allow for sanction of free riders^36^. The agent thus learns a policy that is not readily assigned to a specific philosophy of distributive justice but creatively combines ideas from across the political spectrum.

### Experiment 4

**Fig. 4:**
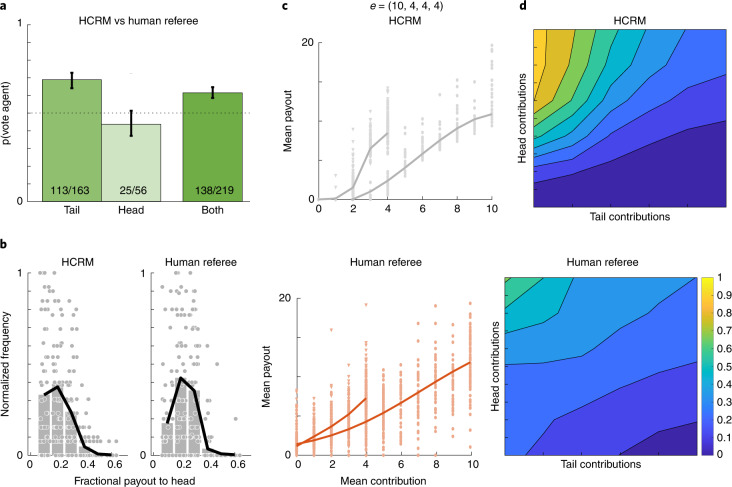
Results of human referee experiment. **a**, Votes for the agent against trained humans. Bars show binomial standard error. **b**, Normalized frequencies of fractional payouts to the head player in 6 equally spaced bins. Each dot at each *x* position represents a single group (*n* = 86); the black line and bars are the average. Note that the HCRM is more willing to offer low payout to the head player than human referees (leftwards skew for left-hand plot). **c**, Empirically observed relationship between contributions and payouts for each HCRM (top) and the human referee (bottom). Each dot is a head player (circle) or average of tail players (triangle) in a single game. The slope mapping contributions onto payouts for the tail player is shallower for human referees. **d**, Empirically observed relationship between payout and head/tail player contributions (‘beach plot’) for HCRM (top) and human referee (bottom) for (10, 4, 4, 4). The colour scale shows the fraction of the public fund allocated to the head player as a function of the head (*y* axis) and tail (*x* axis) player relative contributions.

Finally, we asked whether trained and incentivized human players could have devised a mechanism that was as popular as HCRM. We first recruited 61 previous players and trained them over the course of about an hour to redistribute funds to virtual citizens with a view to maximizing votes (that is, the same training regime as our agent). Human referees earned £2 per vote. Focusing on the (10, 4, 4, 4) condition, which was among those that HCRM found most challenging, we then recruited an additional set of new human players (*n* = 244) who played one game under HCRM and another with trained human referees, in counterbalanced order. These human players strongly preferred HCRM over the human referee (62.4% voted for HCRM, *P* < 0.001). Interestingly, the human players were overall less prone to sanction the head player with low payouts (government × payout sextile interaction, *F*_2,128_ = 5.541, *P* < 0.005, *η*^2^ = 0.125; Fig. 4b) and failed to reward the tail players sufficiently for contributing generously from the little they had (Fig. 4c, bottom) relative to HCRM (top). We show empirically derived beach plots for the human and algorithmic referees side by side in Fig. 4d; they imply that overall, human referees were less responsive to contributions when allocating payouts.

## Discussion

Together, these results thus demonstrate that an AI system can be trained to satisfy a democratic objective, by designing a mechanism that humans demonstrably prefer in an incentive-compatible economic game. Earlier studies have used voting to understand participants’ preferences over contribution thresholds, or exclusion policies in the public goods game^37–39^, but here we used tools from AI research to learn a redistribution scheme from scratch. Our approach to value alignment relieves AI researchers — who may themselves be biased or are unrepresentative of the wider population — of the burden of choosing a domain-specific objective for optimization. Instead, we show that it is possible to harness for value alignment the same democratic tools for achieving consensus that are used in the wider human society to elect representatives, decide public policy or make legal judgements.

Our research raises several questions, some of them theoretically challenging. One might ask whether it is a good idea to emphasize a democratic objective as a method for value alignment. Democratic AI potentially inherits from other democratic approaches a tendency to enfranchise the many at the expense of the few: the ‘tyranny of the majority’^40^. This is particularly pertinent given the pressing concern that AI might be deployed in way that exacerbates existing patterns of bias, discrimination or unfairness in society^41^. In our investment game, we sampled endowment conditions to match plausible real-world income distributions, where the disadvantaged inevitably outnumber the advantaged; hence, for the specific question of distributive justice that we address, this problem is less acute. However, we acknowledge that if deployed as a general method, without further innovation, there does exist the possibility that (similar to real-world democratic systems) it could be used in a way that favours the preferences of a majority over a minority group. One potential solution would be to augment the cost function in ways that redress this issue, much as protections for minorities are often enshrined in law.

Another important point concerns the explainability of our AI-designed mechanism^10^. We deliberately hampered the mechanism designer by not equipping it with activation memory. This means that the mechanism it designed (HCRM) can be transparently described in just two dimensions (rather than, for example, being a complicated nonlinear function of the choice history of different players). Although this level of complexity is greater than the human-generated theories of distributive justice that we use as baselines, it is still possible to verbalize. Encouraging a more interpretable mechanism has at least two advantages. First, it made the agent more transparent to the human players. In fact, in feedback questions (Supplementary Fig. 6), humans deemed the agent to be ‘more transparent and predictable’ than the alternative AI-designed mechanism (rational mechanism) and (perhaps incongruously) the strict egalitarian. Second, the lack of memory has implications for user privacy. Inputs to the agent were designed to be entirely ‘slot equivariant’, meaning that the mechanisms treated each player’s input independently of its ‘slot’ (whether it is player 1, 2, 3 or 4). The agent’s input pertained to the distribution of contributions rather than contributions from individuals themselves. Coupled with the lack of memory, this means that the agent is barred from tracking information about a particular player’s history of contributions within the game.

Our AI system designed a mechanism for redistribution that was more popular than that implemented by human players. This is especially interesting because unlike our agent, human referees could integrate information over multiple timesteps to reward or sanction players on the basis of their past behaviour. However, on average the human-invented redistribution policy tended to reward the tail player insufficiently for making relatively large contributions (from their smaller endowment) to the public purse and was less popular than that discovered by HCRM. Humans received lower volumes of training data than HCRM, but presumably enjoyed a lifetime of experience with social situations that involved fair and unfair distribution, so we think they represent a strong baseline, and a proof of concept for AI mechanism design.

One remaining open question is whether people will trust AI systems to design mechanisms in place of humans. Had they known the identities of referees, players might have preferred human over agent referees simply for this reason. However, it is also true that people often trust AI systems when tasks are perceived to be too complex for human actors^42^. We hope that future studies will address this question. Another question concerns whether participants would have responded differently if the mechanisms had been explained to them verbally, rather than learned by experience. A long literature has suggested that people sometimes behave differently when mechanisms are ‘by description’ rather than ‘by experience’, especially for risky choices^43^. However, AI-designed mechanisms may not always be verbalizable, and it seems probable that behaviours observed in such case may depend on exactly the choice of description adopted by the researcher.

Finally, we emphasize that our results do not imply support for a form of ‘AI government’, whereby autonomous agents make policy decisions without human intervention^44,45^. We see Democratic AI as a research methodology for designing potentially beneficial mechanisms, not a recipe for deploying AI in the public sphere. This follows a tradition in the study of technocratic political apparatus that distinguishes between policy development and policy implementation, with the latter remaining in the hands of elected (human) representatives^46^. We hope that further development of the method will furnish tools helpful for addressing real-world problems in a truly human-aligned fashion.

## Methods

### Participants

The study was approved by HuBREC (Human Behavioural Research Ethics Committee), which is a research ethics committee run within Deepmind but staffed/chaired by academics from outside the company. Participants were recruited over an approximately 8-month period from two different crowdsourcing platforms. All participants gave informed consent to participate in the experiment. The task was advertised to users located in the UK and the USA. We did not record identifiers or personal information from participants. Participants who accepted the Human Intelligence Task (HIT) received a link that led them to a game lobby in which they were grouped with three other players. Groups of four players participated in the game live and in interaction with one another. When a response was required, participants had a fixed duration in which to respond (2 min for all screens except voting, which was 4 min), accompanied by a timer that signalled how much time they had remaining. The game advanced only when the player who was slowest to respond had completed the round. Players who timed out were given a warning. Players who timed out twice were removed from the game and replaced with a randomly responding bot (games with missing data were excluded from the analysis). The game took approximately 20–30 min and participants were paid up to £8, consisting of a base and a bonus payment. The precise conversion rate of points earned in-game (return in coins) to the bonus paid at the end of the study varied inversely with the sum of endowments over players. This way stakes were on average equated across games. Data collection and analysis were not performed blind to the conditions of the experiments.

A total of *n* = 4,776 participants took part in Exp. 1–3. The pilot data that were used for training the agent consisted of a further ~4,000 datasets (including some partial datasets where participants timed out). Exclusion lists were used to prevent participants from rejoining the experiments multiple times (however, as the overall data were collected over several months on two platforms, and we did not collect identifiers, it was impossible to be absolutely sure that all participants are unique).

### Investment game

All participants in Exp. 1–3 played 34 rounds of an investment game (3 blocks of 10 rounds and 1 ‘bonus’ block of 4 rounds). On each round, each player was allocated an endowment of 2, 4, 6, 8 or 10 coins (2, 4 or 10 coins in Exp. 1) depending on the endowment condition to which they were allocated, and whether they were designated the ‘head’ or ‘tail’ player, all of which was entirely random (and unrelated, for example, to the order in which they joined the game). In all endowment conditions, there was a single head player who received 10 coins and three tail players who all received either 2, 4, 6, 8 or 10 coins (in the ‘equal’ endowment condition, the distinction between head and tail players is nominal only). Players received their endowment at the start of each round. Each player’s endowment remained the same across all 34 rounds (and they were instructed that this would be the case).

In every round of every block, each player *i* privately chose to divide an integer number of coins (their endowment *e*_*i*_) between a ‘project’ and a ‘private account’ (the contributions made to the project are denoted *c*_*i*_). No player could see the others’ choices at this stage. The ‘project’ was a public fund that received a return on investment (was multiplied by a common productivity factor *r* = 1.6) and was then shared between participants according to some redistribution scheme, allocating a payout *y*_*i*_ to player *i* (see below). Coins allocated to the ‘private account’ were simply retained by participants (with no return on investment). The total return to each player on each round was thus their payout plus endowment minus contribution ${{{y}}}_{{{i}}} + {{{e}}}_{{{i}}} - {{{c}}}_{{{i}}}$.

In the first block of all experiments, participants played 10 ‘tutorial’ rounds with no referee. This meant that funds allocated to the project were distributed equally among all players and there was no further redistribution (see below). In making this choice, we assume that equal redistribution is a ‘default’ position, which the referees subsequently adjust. There were several reasons for this choice, including the importance of illustrating to players that a social dilemma could arise. However, to ensure that this did not influence our results, we ran additional controls (not reported) in which we added a block of libertarian after the ‘no referee’ block. For the conditions we checked, performance was almost identical to under the default referee, and our agent continued to be preferred in all cases.

In blocks 2 and 3, participants played 10 rounds with a referee (or mechanism; one mechanism for each block). The referee(s) redistributed project funds among players according to a specified mechanism, without creating or destroying wealth. The two rival mechanisms were encountered in counterbalanced order. After block 3, participants voted for the mechanism that they preferred. They knew that they would be making this vote (and what it would entail) from the end of block 1, before experiencing the mechanisms. In block 4, the probability of re-experiencing mechanism A (or B) was exactly equal to the fraction of votes that A (or B) received from the 4 players. The choice was thus deterministic if all players voted the same way, and there was no opportunity to vote strategically. Participants then answered seven debriefing questions (see below). Finally, they experienced four rounds of the chosen mechanism (block 4) and proceeded to a bonus screen where they were thanked and informed of their total earnings. Only data from blocks 2 and 3 were included in the analysis of Exp. 2–4 (see below for Exp. 1). We report numbers of participants who chose to vote for either mechanism using binomial tests.

### Detail of experiments

We describe 3 experiments in the main text. All experiments had the same form but the way we present the data is slightly different for Exp. 1 relative to Exp. 2 and Exp. 3.

In Exp. 2 (*n* = 2,508), players experienced the HCRM and either strict egalitarian, libertarian or liberal egalitarian mechanisms in randomized groups (we provide details about all mechanisms below). The order of encounter of the different mechanisms was counterbalanced over blocks 2 and 3. Under the strict egalitarian mechanism, the referee effectively took no action, so that the original earnings and ‘earnings after referee’s actions’ screens looked the same (apart from bar colour). Under libertarian, the mechanism returned to each player a sum that was 1.6× their contribution. We decided to recruit a larger cohort for liberal egalitarian because simulation data (Supplementary Table 2) suggested that this was the highest performing baseline and potentially more data would be required to draw a reliable conclusion about which was preferred (target participant numbers were decided beforehand, and we did not use optional stopping). In Exp. 3 (*n* = 736), players experienced HCRM and RM (trained with rational players; see below for description of these players, and main text for more details about the RM) in counterbalanced order over blocks 2 and 3.

The data described as ‘Experiment 1’ came from a study in which an earlier version of the HCRM competed against libertarian and liberal egalitarian. We present data only from these two baselines (not the earlier version of the agent). The data from strict egalitarian are taken from block 1 (in which there was no referee). Thus, the conditions under which these data were collected are the same as Exp. 2, except that (1) the order of the mechanisms was not counterbalanced, (2) the rival AI-designed mechanism was slightly different and (3) we did not report voting data from this experiment. Our goal here is to illustrate how contributions vary under different mechanisms; in fact, a near-identical pattern of results was replicated in Exp. 2. At this stage we only used three endowment conditions: (10, 2, 2, 2), (10, 4, 4, 4) and equality ((10, 10, 10, 10)).

### Data pre-processing and analysis

Our main analyses focus on data from Exp. 1–3 (*n* = 4,776 total). We have made this large dataset freely available at https://github.com/deepmind/hcmd_dai, along with code for recreating key figures.

In Exp. 1, we plot contributions as a function of mechanism and endowment as described above (Fig. 1c). In Exp. 2–3, data were analysed from blocks 2 and 3, that is from the two blocks of 10 trials in which participants played the game with a referee that was either the HCRM or a rival baseline. We logged or computed various per-block, per-round metrics for each individual player, including (absolute) contributions (*c*_*i*_), relative contributions (${{{c}}}_{{{i}}}/{{{e}}}_{{{i}}}$), (absolute) payouts (*y*_*i*_), relative payouts (${{{y}}}_{{{i}}}/{{{e}}}_{{{i}}}$) and return (${{{e}}}_{{{i}}} - {{{c}}}_{{{i}}} + {{{y}}}_{{{i}}}$), as well as some group-level game metrics including Gini coefficient and surplus (sum of returns/sum of initial endowments over players and rounds). We show contributions and payouts over time for head and tail players under each mechanism in Supplementary Figs. 3 and 4.

Our voting data consisted of 4 binary votes per group *g*, which were either for HCRM or a baseline. We performed a group-level permutation test to assess statistical significance. Our permuted data randomly flipped voting preferences but preserved the covariance among votes within a group (note that statistics from a naive binomial test might be inflated as they are not independently conditioned on participants’ shared experiences of the mechanism). The lowest possible *P* value obtainable given our 10,000 shuffles is *P* < 0.0001 (but we do not report *P* values lower than 1/1,000 to meet journal style requirements).

### Overview of the mechanism training method

Our approach to AI mechanism design consisted of three steps: (1) we trained virtual human players with supervised learning to imitate human play in the investment game using an existing dataset, which we describe as ‘pilot’ data for the main experiments presented here; (2) we trained a vote-maximizing mechanism using a deep RL agent interacting with these virtual players; and (3) we evaluated the mechanism by deploying it with new unseen human participants, together with comparison baselines (see above). This last step yielded new data, which could be used to repeat the steps above, and refine both the virtual players and the mechanism. In the next section, we describe these steps in detail.

### Training virtual players using imitation learning

We drew upon data from previous pilot experiments (see ‘pilot testing’, below) to train virtual human players using imitation learning. All data were collected from human participants playing the 10-round investment game (described above) under a variety of different mechanisms and varying endowment conditions (not equally distributed). While generally we included only games in which all players finished the experiment, the training data included data from a handful of pilot games where one or more players dropped out. However, the modelling excluded the responses from the players that had been replaced with randomly responding bots. For more details, see ‘pilot testing’ below.

We used imitation learning in which virtual players were trained to imitate human play. Virtual human players were deep neural networks. Each network was a simulation of a single player, which received information about all players’ contributions and receipts on the current trial (similar to real human players), and was trained to predict the focal player’s contributions on the next trial. As they were equipped with recurrent memory (long short term-memory (LSTM) units) (37), the networks could potentially learn to use the trial history going back to the start of the game to make this prediction.

The network received the following information as input on each step: each player’s endowment (4 inputs); each player’s previous contribution (4 inputs); each player’s previous contribution relative to endowment (4 inputs); and each player’s payout (4 inputs). Payouts, endowments and contributions were divided by 10 to lie in approximately the same range as relative contributions. These inputs were fed to a linear layer with output size of 64 and tanh nonlinearities, followed by an LSTM with hidden size 16. The LSTM outputs to a final linear layer of size 11 whose outputs coded the unnormalized log-probabilities of a categorical distribution corresponding to the probability of contributing 0, 1, …, 9 or 10 coins. We masked those outputs corresponding to contributions in excess of the endowment allocated to the focal player.

We trained this architecture with back-propagation through time (38) to minimize the cross-entropy between the predictions and the actual contributions, regularized with two additional terms: the entropy of the prediction (with weight 0.1); and the L2 loss on the parameters of the network (with weight 0.00001). The model was implemented in TensorFlow 1 and the architecture was optimized using Adam (39) with learning rate 0.0004 and parameters beta1 0.9, beta2 0.999 and epsilon 1 × 10^−8^. We trained the model by performing 30,000 updates with mini batches of size 512. Training took <6 h without the use of accelerators.

The virtual human player networks were evaluated on a separate hold-out dataset consisting of the contributions of a new group of human players (*n* = 384) that resembled as closely as possible the final conditions under which we expected to evaluate the HCRM. We swept over network hyperparameters (including layer widths, number of LSTM units, learning rate and regularization types) to minimize validation loss.

### Voting model

We learned in piloting that human players’ votes are most strongly predicted by the relative payouts (${{{y}}}_{{{i}}}/{{{e}}}_{{{i}}}$) they receive under one mechanism or another. We thus used this variable as the basis for virtual player voting. Each virtual player’s probability of voting for mechanism A rather than the rival mechanism B was ${{{p}}}\left( {{{\mathrm{A}}}} \right) = {{\Phi }}\left[ {{{{\mathrm{rpay}}}}^{{{\mathrm{A}}}} - {{{\mathrm{rpay}}}}^{{{\mathrm{B}}}}} \right]$ where ${{{\mathrm{rpay}}}}^{{{\mathrm{M}}}}$ is the sum of relative payouts obtained under mechanism M and ${{\Phi }}\left[ \cdot \right]$ is a logistic function with slope *s*. We set *s* to be 1.4, but similar results were obtained (in terms of mechanism policy, see below) under a wide range of values (see below).

### Mechanism designer: problem definition and setup

We call the neural network used to design the mechanism the mechanism designer and use the term human-compatible mechanism (HCRM) to refer to the mechanism it designs, which was obtained only after training has converged. It used RL to learn a function that mapped observations (game states generated through interaction with virtual players) onto redistribution weights (a variable that determines which player gets what fraction of the project fund). We chose a Graph Network-based architecture that is equivariant to permutation in the ordering of participants and trained it on simulated 10-round investment games, with the goal of maximizing the cumulative voting probabilities of the virtual players against a carefully chosen alternative mechanism (see below). After training for 10,000 steps, the network parameters were frozen, and the function was exported in a way that allowed ready implementation in a human testing setting.

#### Network architecture

Inputs to the network were the endowments, contributions and relative contributions (that is, contribution–endowment ratios) for the current round, for all four participants (12 inputs per round). The network’s output was 4 numbers that were passed through a softmax function (that is, so that they are positive and sum to 1) to generate the redistribution weights for each player. When we state that the network has no memory, we mean that (1) it does not receive historical information about contributions or payouts as inputs; and (2) it does not have recurrence, that is, network states are not passed between timesteps. Note, however, that the mechanism could infer implicitly the number of rounds if human/virtual player policies vary their contributions between different timepoints within the block.

We organized the network’s observations in a fully connected directed graph $({{{u}}},{{{V}}},{{{E}}})$ where each player was represented as a vertex ${{{v}}}_{{{\mathrm{k}}}} \in {{{V}}}$. Directed edges *e*_s,r_ connecting *v*_s_ and *v*_r_ had empty initial attributes, and the input global attribute vector *u* was empty. Computations in Graph Networks start by updating the edge attributes, followed by the node attributes and finally global attributes. In particular, directed edge attributes were updated with a function ${\upvarphi}_{{{e}}}$ of the input edge attribute, the sender and receiver vertex attributes, and the global attributes vector: ${{{e}}}_{{{{\mathrm{s}}}},{{{\mathrm{r}}}}}\prime = {\upvarphi}_{{{e}}}({{{e}}}_{{{{\mathrm{s}}}},{{{\mathrm{r}}}}},{{{v}}}_{{{\mathrm{s}}}},{{{v}}}_{{{\mathrm{r}}}},{{{u}}})$; vertex attributes were updated as a function ${\upvarphi}_{{{v}}}$ of the input vertex attributes, the sum of all updated edge attributes that connect into *v*_r_, and the global attributes vector: ${{{v}}}_{{{\mathrm{r}}}}\prime = {\upvarphi}_{{{\mathrm{v}}}}({{\Sigma }}_{{{\mathrm{s}}}}{{{e}}}_{{{{\mathrm{s}}}},{{{\mathrm{r}}}}}\prime ,{{{v}}}_{{{\mathrm{r}}}},{{{u}}})$; finally, the global attributes vector was updated with a function of the input global attributes, and the sum of all updated edges and vertices: ${{{u}}}\prime = {\upvarphi}_{{{u}}}({{\Sigma }}_{{{{\mathrm{s}}}},{{{\mathrm{r}}}}}{{{e}}}_{{{{\mathrm{s}}}},{{{\mathrm{r}}}}}\prime ,{{\Sigma }}_{{{\mathrm{k}}}}{{{v}}}_{{{\mathrm{k}}}}\prime ,{{{u}}})$. We note that the same functions ${\upvarphi}_{{{e}}}$, ${\upvarphi}_{{{v}}}$ are used to update all edges and nodes in a graph, and that both the input and output of Graph Networks are directed graphs, so these modules can be used in sequence.

The mechanism designer’s policy network architecture consisted of two Graph Networks (GNs) that processed the observation in sequence. In the first GN, we implemented all of ${\upvarphi}_{{{e}}}$, ${\upvarphi}_{{{v}}}$ and ${\upvarphi}_{{{u}}}$ as distinct linear layers with 32 output units and tanh activation functions. In the second GN, we implemented ${\upvarphi}_{{{\mathrm{e}}}}$ as a linear layer with 32 output units and tanh activation function, and ${\upvarphi}_{{{\mathrm{v}}}}$ as a linear layer with a single output unit. We normalized the vertex outputs with a softmax across players, thus obtaining the redistribution weights; ${\upvarphi}_{{{u}}}$ was ignored in the second GN.

#### Training algorithm

To train the mechanism, we used an estimator drawn from a framework known as Stochastic Computation Graphs (SCG)^30^, which approximates the gradients of the vote-maximization objectives with respect to the network’s policy parameters. We trained the mechanism designer iteratively with 10,000 updates using the RMSProp algorithm to optimize the policy, with the following parameters: learning rate 0.0004; epsilon 1×10^−5^; decay 0.99; and no momentum. On every update, we simulated two batches of 512 games with 10 rounds per game. We divided the batches in groups of 64 episodes with consistent endowments, leading to 8 possible endowments: the head player received 10 coins, and the tail players received 2, 3, 4, 5, 6, 7, 8 or 10 coins (using a broader range of tail player endowments helped avoid overfitting).

On every round, the game unfolded as described above for human players, except that the contributions were dictated by the virtual human players. In the first block, the redistribution policy was decided by the mechanism designer under training, and in the other it was played by an alternative planner (which was the winner of the metagame, defined by ${{{w}}} = 1,{{{v}}} = 1$; see section on ideological manifold). We paired episodes from these two batches to obtain 2,048 votes (512 pairs of episodes, 4 players) given our model of human voting. The objective that the HCRM aimed to maximize was the sum of votes across players, averaged across episodes.

Note that during training of the mechanism, we did not feed in the human data to predict player contributions (that is, ‘teacher forcing’). Furthermore, the payouts observed by the virtual players depended on mechanism policies that may lie outside of the human data, thus requiring the virtual players to generalize beyond the training dataset.

Having defined the observations as well as the objective that we wished to maximize, we estimated the policy gradient, that is, the gradient of the objective (the average number of votes) with respect to the policy parameters (the weights of the graph network) by turning to the SCG framework. We note here that most of the computation in the investment game is differentiable (including the policy as implemented by the HCRM), with the virtual human players policies, whose action space is discrete, being the only exception. The SCG framework generalizes the policy gradient theorem and allowed us to obtain a low-variance estimator of the policy gradient by auto-differentiating through the environment and mechanism policy, while compensating for the non-differentiable operations (the discrete contributions of the players). The surrogate objective for the policy gradient was as follows:

$${{{S}}} = {{{J}}} + \bot ({{{J}}}) \times {{\Sigma }}_{{{\mathrm{i}}}}{{\Sigma }}_{{{{t}}} = 2}^{10}{{{\mathrm{logp}}}}( \bot ({{{c}}}_{{{\mathrm{i}}}}^{{{\mathrm{t}}}})),$$

where *S* is the surrogate objective, *J* is the objective we wish to maximize per episode (the expected number of votes) and ⊥ is the stop-gradient operation. Note that for the second term, the gradient can only flow through the parameterization of the log-probability of the player’s policy. Note also that the contributions of the first round are removed from the equation since they do not depend on the mechanism’s parameters. In practice, additionally, we chose to mean-center *J* within a batch because this is known to reduce the variance of the gradient estimator.

In Supplementary Information, we include further details that provide (1) a detailed description and illustration of the game, (2) the voting procedure, (3) debriefing, (4) determinants of voting analysis, (5) beach plots, (6) the ideological manifold, (7) rational players, (8) the metagame, (9) pilot testing, (10) human referee experiments and (11) theoretical analysis of the game.

### Reporting summary

Further information on research design is available in the Nature Research Reporting Summary linked to this article.

## Supplementary information

Supplementary Information Supplementary methods, results, Tables 1 and 2, and Figs. 1–10.

Reporting Summary

## Data Availability

All human data is available at https://github.com/deepmind/hcmd_dai.

## Code availability

Code for reproducing figures is available at https://github.com/deepmind/hcmd_dai.
