# Publisher Correction: Human-centred mechanism design with Democratic AI

**DOI:** 10.1038/s41562-022-01444-1

**Published:** 2022-08-12

**Authors:** Raphael Koster, Jan Balaguer, Andrea Tacchetti, Ari Weinstein, Tina Zhu, Oliver Hauser, Duncan Williams, Lucy Campbell-Gillingham, Phoebe Thacker, Matthew Botvinick, Christopher Summerfield

**Affiliations:** 1 Deepmind, London, UK; 2 Department of Economics and Institute for Data Science and Artificial Intelligence, University of Exeter, Exeter, UK; 3 Gatsby Computational Neuroscience Unit, University College London, London, UK; 4 Department of Experimental Psychology, University of Oxford, Oxford, UK

**Keywords:** Science, technology and society, Economics

Correction to: *Nature Human Behaviour* 10.1038/s41562-022-01383-x, published online 4 July 2022.

In the version of this article initially published, author Jan Balaguer’s name was presented as Balaguer Jan. The error has now been corrected in the HTML and PDF versions of the article.

