# Supplementary material for: Human-centred mechanism design with Democratic AI
Source: Nat Hum Behav. 2022 Jul 4;6(10):1398–407. doi: 10.1038/s41562-022-01383-x (PMC9584820; doi:10.1038/s41562-022-01383-x)
Supplement: Supplementary file 1 — Supplementary methods, results, Tables 1 and 2, and Figs. 1–10. [file 41562_2022_1383_MOESM1_ESM.pdf]

nature portfolio

https://doi.org/10.1038/s41562-022-01383-x

Supplementary information

Human-centred mechanism design with Democratic AI

In the format provided by the authors and unedited

Supplementary methods for:

Human-centered mechanism design with Democratic AI

Authors: Raphael Koster[1]†, Jan Balaguer[1]†, Andrea Tacchetti[1], Ari Weinstein[1], Tina Zhu[1], Oliver Hauser[2], Duncan Williams[1], Lucy Campbell-Gillingham[1], Phoebe Thacker[1], Matthew Botvinick[1,3] and Christopher Summerfield*[1,4]

Affiliations:

[1] Deepmind, London, UK

[2] Department of Economics and Institute for Data Science and Artificial Intelligence, University of Exeter, Exeter, UK

[3] Gatsby Computational Neuroscience Unit, University College London, London, UK

[4] Department of Experimental Psychology, University of Oxford, Oxford, UK

*Corresponding author. Email: csummerfield@deepmind.com. ORCID 0000-0002-2941-2653

1. Detailed description and illustration of the game

Participants began the task with instructions and a tutorial. Players were initially told that they were playing an investment game and could “earn points depending on both [their] own decisions and on the decisions of others” and that they would "receive a base payment for completing the task but could also get a bonus, “depending on how many points [they] earn”. They then underwent a demonstration round with identical structure to each of the real rounds in the game, except that their choices were not logged.

On every round each player was allocated an endowment consisting of an integer number of coins and decided to contribute each coin to a “project” or a “private account” by pressing buttons with those corresponding labels (see image below). After each response a coin disappeared from the endowment row and appeared below the button that had been pressed (project or private account, see below). When all coins had been allocated, the “submit response” button became available, and participants pressed this to complete the trial. The screens below show an example for a player whose endowment was 10 coins:

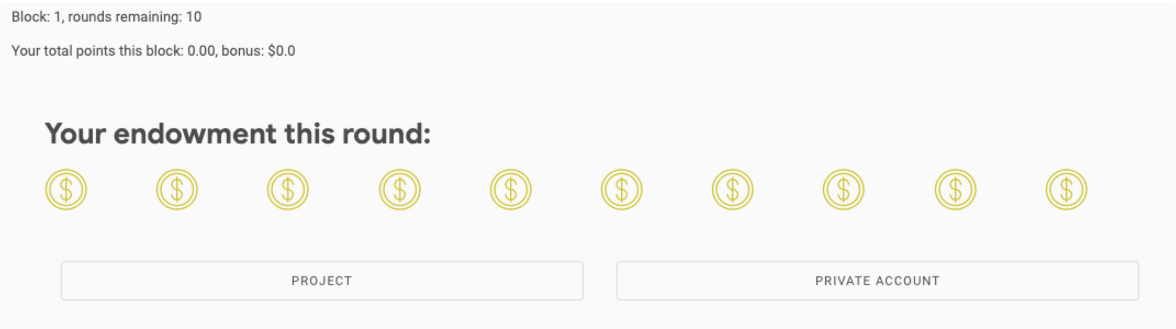

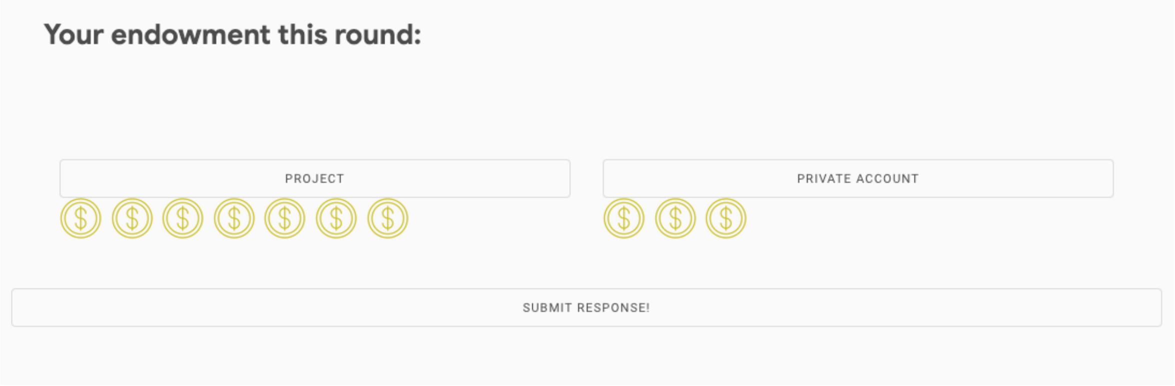

After all players had completed the round by allocating their full endowment of coins and pressing “submit response” they viewed a results screen (image below). On all rounds, three panels were shown (Supplementary Figure 2). The upper panel was a bar graph that showed contributions to the private

account and project made by each of the 4 players, including the focal player, both numerically and in the form of a bar graph. Players were denoted with a distinctive icon. The bar graph was always scaled so the y-axis ranged from 0-10 coins.

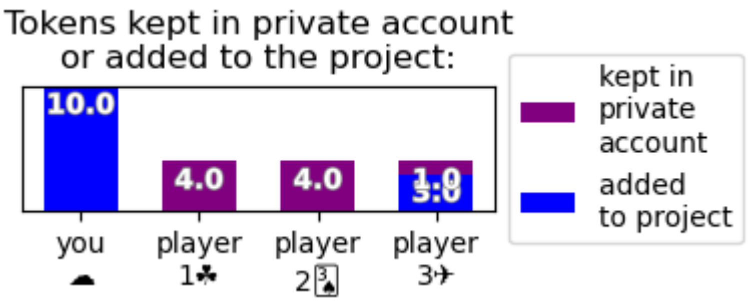

The second panel differed according to whether players were in the training game (block 1) or the test or bonus games (blocks 2-4). In the training game, there was no “referee”. An example of the screen shown in this block is given below. It reveals the “project earnings” for each player (this is called “payout” in the main text and denoted y_i for player i). In the example, 10 coins have been allocated to the project (6 from the focal player, and four from player 3). These are increased to 16 coins following the multiplication factor of r = 1.6. Note that when there is no referee, distribution is always equal between players, which is identical to the mechanism “strict egalitarian”.

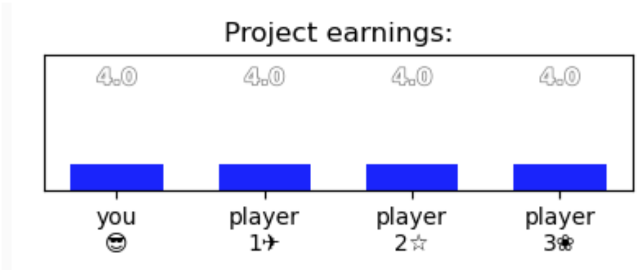

In blocks 2-4 there was a “referee”, which was the AI-designed mechanism HCRM or a rival baseline. In either case, participants viewed a screen that looked like this:

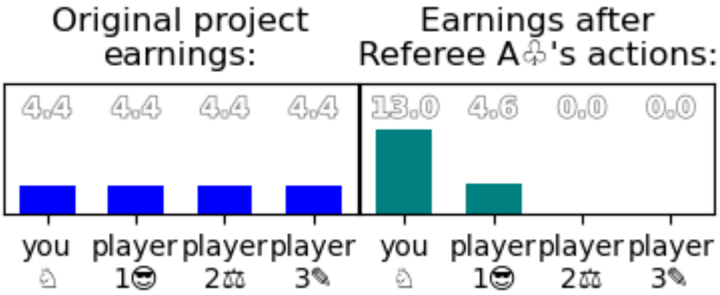

The screen on the left is the same as for block 1, except that the title "project earnings" becomes "original project earnings". The referees' actions were framed as an intervention on a default distribution, which is identical to 'strict egalitarian'. On the right hand side, players saw an additional panel that shows how these earnings have been redistributed among players by the referee, hence 'after' the referee's action. The referees were referred to as "A" and "B" and also identified by a unique color and symbol. All earnings before and after redistribution were displayed to one decimal place as in the example (note that although contributions were constrained to integer values, payouts were not) The bar color for the rightmost panel varied according to the referee identity.

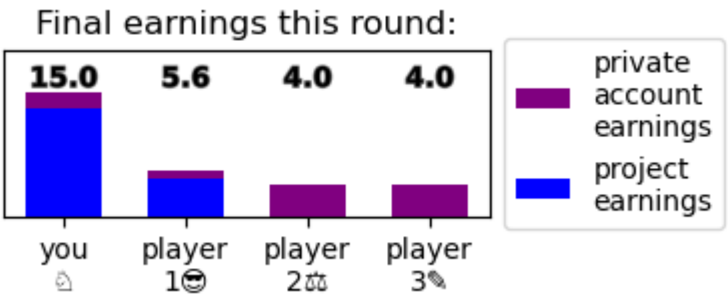

In all blocks, the final panel of the results screen gives participants a final overview about how much return was obtained by each participant, divided earnings returned from the project and by what was kept in the private account.

2. Voting procedure

An illustration of the voting screen is shown below:

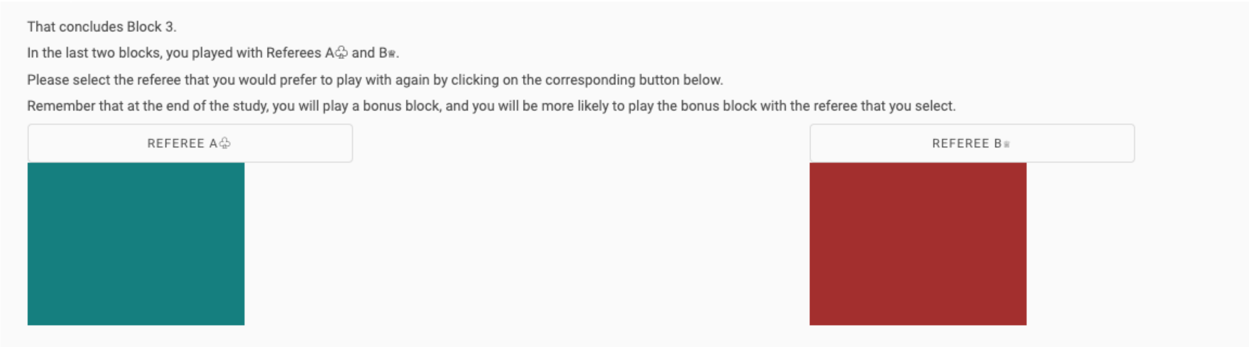

Participants were instructed to make a binary choice between the two referees (each marked with a different letter, symbol and color which matched those seen during the results screen for each mechanism). It was highlighted to participants that their choice will affect the likelihood of playing the last block with the referee they chose. After their selection, a button appeared that asked participants to confirm their choice.

3. Debriefing

After the vote (but before block 4) participants answered 7 binary debriefing questions:

1. Which referee will lead to everyone doing better?
2. Which referee will lead to you individually doing better?
3. Which referee will lead to the majority of players doing better?
4. Which referee will be fairer?
5. Which referee will be best at encouraging collaboration?
6. Which referee will be more permissive or lenient?
7. Which referee will be more transparent or predictable?

Participants responded by checking a radio button next to either “referee A” or “referee B” following each question. The results of this debriefing questionnaire are shown in Supplementary Figure 6.

4. Determinants of voting

We conducted an analysis to understand the determinants of voting using logistic regression. Results are shown in Supplementary Figure 2.

For votes in Exp. 2-3 we constructed a regression model with the following form:

$$p\left(vote^{HCRM}\right) = \Phi[\beta_0 + \beta_1 \cdot rpay \ + \beta_2 \cdot apay + \beta_3 \cdot cont]$$

Where rpay, apay, cont, and gini are relative variables that encode an aggregate quantity (over 10 rounds) under the HCRM minus that same quantity under the rival mechanism (rpay is payout divided by endowment for the focal player; apay is absolute payout to the focal player; and cont is the sum of contributions of all group members. We tried other regressions that included Gini coefficient and/or payout to other players, but these fit less well. The logistic function is denoted $\Phi[\cdot]$. Predictors were standardised before being entered into the regression, so the y-axis in Supplementary Figure 2 shows units of standard deviation.

We report statistics for β_0 to β_3 in an additional table.

5. Beach plots

In order to visualize the HCRM, we plotted the fraction of payouts that were allocated to the head player as a function of an exhaustive set of possible contributions (relative to endowment) made by simulated head and tail players. Note that because the HCRM is time-invariant there exists only a single such surface (not one per timepoint). To aid visualization, we averaged across the contributions of the tail player, reducing a four-dimensional matrix to just two dimensions. The plot in Figure 3a (main text) thus shows the average fraction of returns to head player (1-tail player) for each combination of head and (average) tail contributions. In the Supplementary Figure 8 we show equivalent plots for which the dependent measure is the return to either the head or tail player.

6. The ideological manifold

We defined a space of mechanisms that spanned strict egalitarian, libertarian, and liberal egalitarian schemes as follows. First, we assumed that the payout to player i is an admixture of their own (absolute) contribution c_i and the average of other players c_{-i} so that

$$y_i^{abs} = r[w \cdot c_i + (1 - w) \cdot c_{-i}]$$

where w is the mixing parameter. Thus, for example, if $w = 1/k$ (w = 0.25 for our case of 4 players) then y_i^{abs} will be equal for all players. But we can also define a space of relative mechanisms:

$$y_i^{rel} = r\left(\frac{C}{P}\right)[w \cdot \rho_i + (1 - w) \cdot \rho_{-i}]$$

where $\rho_i = c_i/e_i$ is the ratio of contribution to endowment for player i, where c_{-i} and ρ_{-i} are respectively the average contributions and ratios from players other than i, and C and P are the sum of contributions and ratios across all players. We can then define a full space of baseline mechanisms defined by both w and v as a mixture of absolute and relative redistribution:

$$y_i = v\left(y_i^{rel}\right) + (1 - v)(y_i^{abs})$$

Thus if $w = 1$ and $v = 0$ then payout is exclusively driven by y_i^{abs} and thus reduces to $y_i = r \cdot c_i$, which is the libertarian mechanism. Alternatively, if $w = 1$ and $v = 1$ then payout is exclusively driven by y_i^{rel}, and thus depends on ρ_{-i} which is the ratio of contributions to endowment, which is the liberal egalitarian mechanism.

We visualized the space of mechanisms defined by mechanism parameters v and w (see below and main text for details) in terms of the relative payout they gave to head and tail players under each endowment (in the main text we show this visualization for endowment [10, 2, 2, 2] in Figure 1b). We used simulated data in which the behavior of virtual human players was unrolled in repeated games of 10 trials. In each game, the payouts were determined by one of 100 mechanisms (each defined by a parameterization of v and w linearly sampled in 10 bins from 0 to 1; see main text and below for more details) under the 5 endowment conditions. This yielded 10 average relative payouts for both head and tail players per mechanism and endowment [20 features] for 500 mechanisms [5 endowment100 instances]. We used multidimensional scaling (MDS) to reduce the dimensionality from 20 to 2 dimensions and plotted the 100 mechanisms for endowment condition [10, 2, 2, 2] all together (Figure 1b). Other endowment conditions are shown in Supplementary Figure 9.

7. Rational players without human data

We also built rational players that learned with gradient descent to maximize their return within a single block (rather than learning to imitate human play). We assume that these players have full information about the environment and exact knowledge of the redistribution mechanism at each point in training. Each rational player was also characterized by two free parameters: a learning rate, sampled from a Gamma distribution ($k = 3$, $\theta = 1$); and a initial generosity, sampled from a Normal distribution ($\mu = 0$, $\sigma = 1$). These parameters were chosen both to match the previous literature and to facilitate convergence near the end of the block (10 rounds). We also trained mechanisms based on different parameters

(Gamma $\theta \in \{1,3\}$, Normal $\sigma \in \{1,3\}$) and performed comparisons between the rational player parameters that the mechanism was trained or evaluated on. Overall, we found that the mechanisms trained with the selected parameters generalized numerically better to other rational player parameters (see supplementary table ST2).

Each round, the rational player performed gradient ascent to maximize their immediate reward with respect to their generosity, assuming that other players' contributions were constant (i.e. independent gradient ascent).

The equations describing the updates of the rational players are as follows:

$$\alpha_i \sim \Gamma(k = 3, \theta = 1)$$
$$g_i{}^1 \sim N(\mu = 0, \sigma = 1)$$
$$c_i{}^t = e_i/(1 + \exp(-g_i{}^t))$$
$$g_i{}^{t+1} = g_i{}^t + \frac{\partial}{\partial g_i{}^t}[y_i{}^t + e_i - c_i{}^t]$$

where i is an index corresponding to each player, $g_i{}^t$ is an intermediate variable that we call "generosity", and α_i is the learning rate at which contributions were updated. Note that the derivative in the last equation depends on everyone's contributions, the scaling factor r of the investment game and the mechanism that redistributes the payouts. In practice, we automatized the computation of this derivative with back-propagation. So as to not disadvantage them unduly, we allowed the rational players to vote according to the same human-derived policy as the virtual human players that were used to train HCRM.

8. Metagame (or round robin tournament)

We trained the mechanism designer to maximize the votes it is expected to receive in an election against an alternative mechanism. We call this a "meta-game". We selected the alternative mechanism by simulating round-robin elections between candidate mechanisms defined by linearly sampled values of v and w (see ideological manifold section for details of how these parameters are used to define mechanisms). We used virtual human players and selected the mechanism that won the highest number of votes against all opponents over games of 10 rounds (we found no Condorcet cycles in our round-robin elections) to function as the alternative mechanism during training. The expected number of votes received by each mechanism was estimated from a total of 4096 independent blocks. The winning mechanism was defined by $v = 1$ and $w = 1$, which corresponds to liberal egalitarian, and which we subsequently implemented as one of our baselines. Results of the round-robin tournament are shown in Table S2.

9. Pilot testing

The data used for training used a variety of data from different experiments (n > 4000, not including data from Exp1-3), all similar in form to the experiments presented above. The games covered a range of different endowment settings and mechanisms, including earlier incarnations of the HCRM. In a subset of the training data participants played 4 blocks with referees and voted twice. Overall, the training set contains 4809 blocks, of which 966 were played under equal endowments, 326 under [10, 4, 4, 4], 322 under [10, 2, 2, 2], 1271 under [10, 5, 4, 3], 324 under [10, 8, 5, 3], and 300 under [10, 9, 8, 3]. For the remaining 1300 pilot participants, the endowment was randomly selected between 1 and 10. Note that within any given block, we only model data from participants who did not time out on any trials (i.e. for whom there are 10 contributions in that block). However, the training data (unlike the validation dataset or Exp. 1-3) contains blocks in which participants dropped out of the game and were replaced with a randomly responding bot (2491 blocks contained all four players, the other blocks contained one or more player who dropped out).

The additional validation dataset contained 288 blocks covering the same conditions as Experiments 2-4. Note that this way the validation set tested a degree of generalization to the [10, 6, 6, 6] and [10, 8, 8, 8] conditions which were not included in the training data.

10. Human referee experiments

To test whether humans could design a similarly successful mechanism, we implemented a version of the game involving four players and an additional participant who takes the role of the referee and redistributes the funds on every round. In what follows we use the term “player” to refer to the participants who receive the endowment on each tround, and “referee” to the participant who redistributes the public fund back to the players.

Endowment condition

In this new experiment, we focussed on the [10, 4, 4, 4] endowment setting. This choice was made for several reasons. Firstly, [10, 4, 4, 4] and [10, 2, 2, 2] were the endowment conditions that (on average against all other competing mechanisms) the agent found most challenging. However, [10, 2, 2, 2] is a less interesting case because the tail player has a highly restricted action space (can only contribute 0, 1 or 2 coins), and simulations with virtual citizens show that liberal egalitarian is optimal in this condition. By contrast, in [10, 4, 4, 4] the agent has to carefully partition funds between the head player (who has the most power to fund the public purse) and the tail players (who collectively have the most

votes). We thus thought that it presented an interesting challenge for our agent to attempt to beat human referees in this endowment condition.

Task (player perspective)

From the perspective of the four players, the new game was identical to the setting with two algorithmic referees. After playing a game under the egalitarian baseline, the players played two games, one with the human referee and one with HCRM. As before, on each of 10 rounds, they made contributions to the private or public funds, and received back any redistributed funds. They then voted, and experienced 4 trials of the referee (human or HCRM) that won the vote.

Unlike HCRM, human referees do not issue decisions instantly, and so we additionally introduced a mechanism to equate the timing of redistribution decisions between HCRM and the human referee, to avoid the possibility that an automated mechanism is preferred because it is faster (thus increasing participants payouts per hour). To rule out this possibility we sampled HCRM reaction times from reaction times recorded in a pilot human sample. In practice, on average these sampled times were slower than those issued by human participants in the main experiment (mean (standard deviation): 40.36 (20.74) vs. 33.14 (14.1) seconds; $t_{60} = 2.2$, $p < 0.02$). Whilst we think it likely that participants would have preferred the faster referee, to test whether voting decisions depended on timing, we correlated RTs of human referees to their percentage votes obtained (out of all players that completed the game), finding no relationship (Pearson's $r_{60} = 0.15$, $p = 0.24$). In combination, these two factors make it unlikely that the results we obtained were driven by any differences in RTs.

Referee Recruitment. We wanted to ensure that participants who take the role of the referee were familiar with the task and interface, so that they understood the incentives and dynamics of the game, allowing them to implement the redistribution policy that they think will maximise votes. Thus, to be eligible to participate as referee, participants must have taken part in a previous version of the study (over the past ~12 months) in the role of a citizen.

Referee Training. To introduce referees to the basic game mechanics and their role, they first completed a detailed tutorial with instructions on the game worked, what their role would be, and how to redistribute. They then performed a quiz with questions about what they had just learned. Subsequently, they completed 3 blocks of 10 rounds playing with virtual players. At the end of each block, players were informed how many votes they received from the virtual players, virtual player votes were determined in the same way as when training HCRM (by simulating games involving the human allocations and those from HRCM with and calculating the votes of virtual citizens based on this comparison; note that this process was not visible or known to the human referees). This training procedure that the referees underwent thus precisely mirrors the training procedure of the HRCM,

except that human referees were only exposed to 3 training episodes. Additionally, prior to performing the main task, referees were again instructed and then performed the quiz, before completing a shorter (5-trial) version of the training (again with virtual players) to refresh their familiarity with the interface. Overall, the training session was completed by participants in between 30 and 60 minutes. Only participants who completed the training were able to sign up for the actual task that was conducted on the same or next day.

Quiz. To verify that referees paid attention and understood the basics of the game, they were asked to answer 14 four-way multiple-choice questions about game scenarios. Each question involved plots reporting some aspect of a round (e.g. the distribution of public contributions over players as a bar plot). They were required to answer questions such as: ‘How much did player 4 receive from the referee?’ or ‘How many coins were available to redistribute for the referee?’. After each question participants received feedback on whether they got the question correct or not. In the event of an error, they were informed of the right answer and verbally oriented to the relevant portion of the plot. For example: 'Player 4 received a total of 6.0 coins! You can see this in the most bottom panel. It displays the final earnings. Participants performed the quiz twice with questions in random order, once after instructions for the training and once after instructions for the refresher task. By the time of the refresher, referees scores 94.6% on average (minimum score 71.4%).

An example quiz screen is shown below.

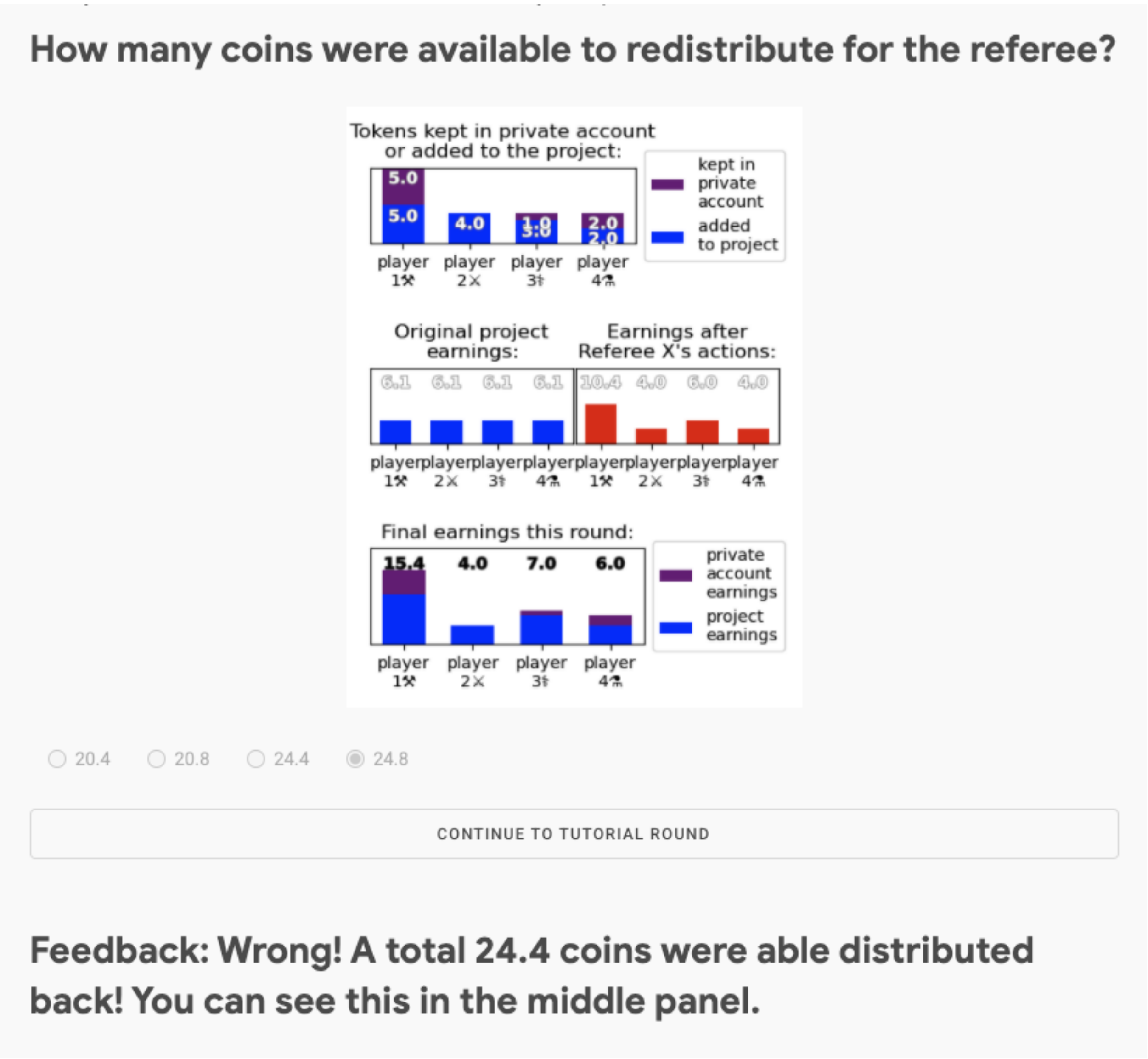

Interface

During the experiment, human referees saw the following interface. It displays how much each player contributed and allows the referee to control the fraction of the pool each player will get as payout in the current round.

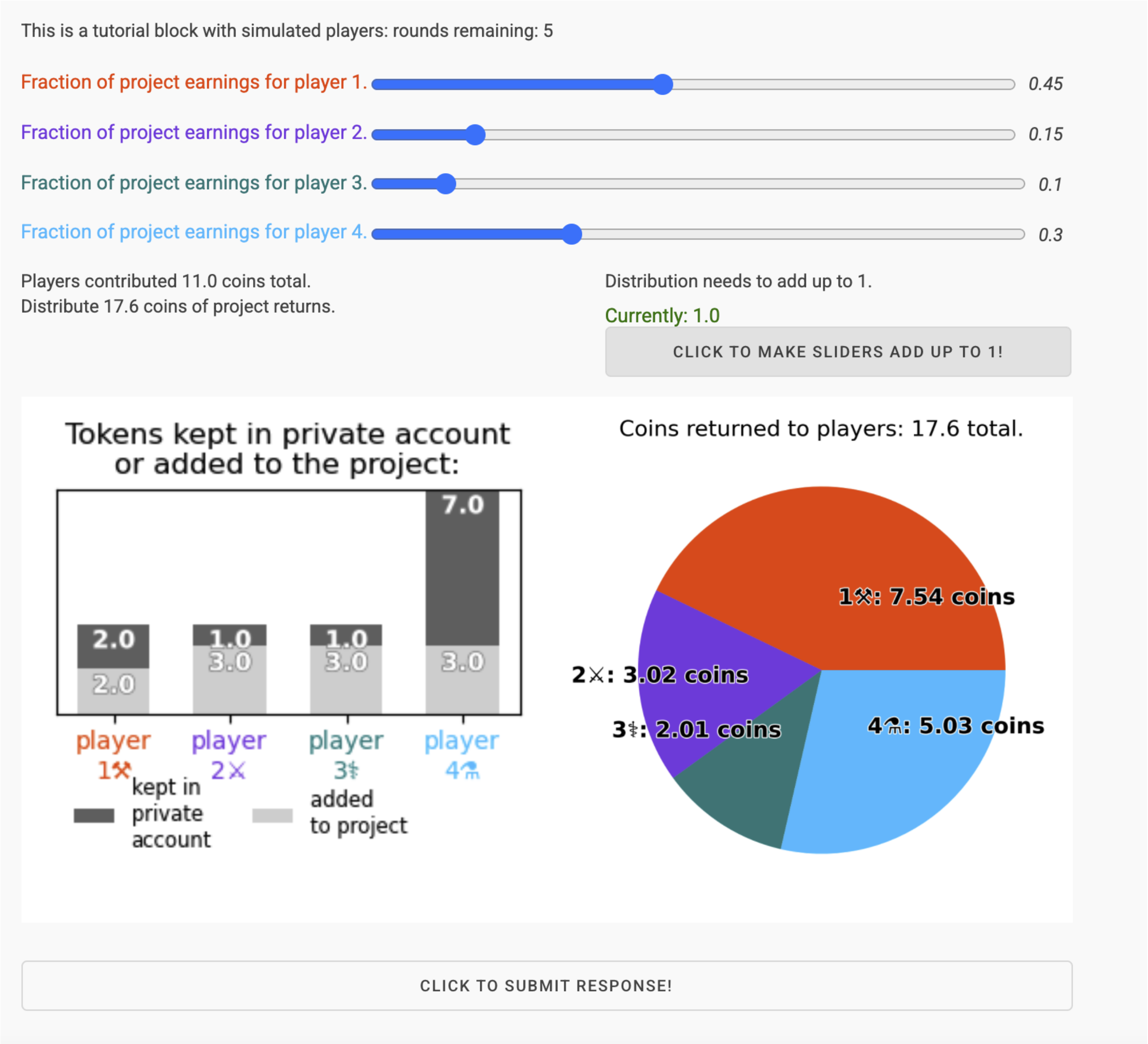

Data Analysis

We analysed 61 games in which the human referee completed the task. These included some games in which players dropped out during the experiment (and were replaced as described previously). This resulted in a dataset with 219 votes cast by players (43 games with 4 votes cast, 13 games with 3, 3 games with 2 and 2 games with 1 vote cast). Our primary analysis is of the fraction of votes received by HCRM relative to the human referee. We conduct statistics on the votes of all players regardless of whether their co-players dropped out or not. However, we note that of the 172 votes cast in games where all players finished ($n = 43$), the players also preferred the HRCM on 62.2% of votes ($p < 0.002$, Binomial test).

We conducted several analyses aimed at revealing differences between the redistribution policies of the human and HCRM. Firstly, we plotted the distributions of fractional payouts to the head player

$payout_{head}$ / ($payout_{head}$ + $payout_{tail}$) under the human and algorithmic referee in six equally spaced bins. An ANOVA revealed that there was an interaction between sextile and referee, driven by a greater tendency for HCRM to make smaller payouts to the head player (Figure 4b). Secondly, we plotted how the payout to head and tail (average) players in each game depended on their contribution (Figure 4c). Finally, we constructed empirical "beach plots" for HCRM and human referees. Note that whereas for algorithmic referees we can probe their response to all possible combinations of contributions by the head and tail player, for human referees we are limited to those that occurred in the actual games (and so beach plots may look slightly different to those shown in Figure 3a). For comparability, we calculated both beach plots in the same way, so the HCRM plot differs slightly from that in Figure 4.

11. Theoretical analysis of the game

We studied the properties of the game in more detail using simulations. We used a combination of analytic and Monte Carlo approaches to estimate the reward which should accrue to a player confronted with a single round of a game involving k players and scaling factor r, under a mechanism defined by v and w.

First, we consider the space of mechanisms that consider only the absolute contribution. If the mechanism exists on the ideological manifold with $v = 1$ then the total reward V to each player is:

$$V_i^{abs} = e_i - c_i + r(wc_i + (1 - w)c_{-i}$$

where r is the growth factor, w defines the mechanisms, and c and e are the contributions and endowments respectively. From this we can compute where on the manifold the derivative of reward with respect to contribution is positive, and this is found at:

$$w > \frac{1}{r}$$

In other words, if the manifold parameter $w > 1/r$ then the Nash equilibrium (NE) is for everyone to contribute the full endowment (independently of number of players and endowment). On the other hand, if $w > 1/r$ then the game is a social dilemma, and the NE is for no one to contribute (pareto deficient).

In the case of $v < 1$ the incentives of each player depend on the endowments and contributions as well as the mechanism and game parameters. The answer is therefore more convoluted and harder to derive by hand. We provide insights for them in simulation.

The total reward accruing to each player will be given by a mixture of the rewards from absolute and relative components:

$$V_i = vV_i^{abs} + (1 - v)V_i^{rel}$$

Where the absolute component is as defined above, and the relative component is

$$V_i^{rel} = e_i - c_i + \frac{rC}{P}(w\rho_i + (1 - w)\rho_{-i}$$

Where ρ is the relative contribution given by c/e and C and P are the sum of contributions and ratios across all players as above.

We can approximate the Nash Equilibrium by initializing the contributions c_i and iteratively updating them in the direction of the gradient $\frac{\partial V_i}{\partial c_i}$ using a tensor programming library (we used JAX). Assuming optimisation converges, the subsequent contributions should be a good approximation of the Nash Equilibrium in that no player has an incentive to change their contributions. This simulation can therefore be used for making predictions about human behaviour near the end of the episode (once participants were able to infer the mechanism policy as well as the contributions from other players).

The result of our simulation is therefore a function:

$$c_{1\ldots k} = \mathrm{GetApproxNash}(e_{1..k}, r, v, w)$$

One implementation detail to add is that, since contribution c_i is bounded in the range [0, e_i], we reparametrize it in logits to perform gradient descent (and then invert back into unit range).

We used this function to explore the relative contribution under the Nash Equilibrium for a range of different values of w, v, r and k (see Supplementary Figure 10).

Supplementary Text. Potential limitations of Democratic AI.

Our work describes a recipe for training an AI system to design a social or economic mechanism for humans. Our success metric is that humans, having experienced the mechanism, will vote for it in a majoritarian election. Our work thus offers a practical way to deal with issues of value alignment in AI research, in that maximizing human preferences (as expressed through votes) is directly set as the agent's goal (it is part of the cost function). However, we acknowledge that there are both practical and conceptual challenges associated with this approach. We use this supplementary section to highlight potential limitations of our approach, and how they might be overcome in future work.

The first and most important point is that our work should not be interpreted as advocating for a form of "AI government", whereby autonomous agents make policy decisions without human intervention (32, 33). We propose Democratic AI as a research pipeline for discovering social or economic mechanisms that humans may prefer, but it does not imply in any way that final decisions over deployment should be ceded from human to agent. This follows a tradition in the study of technocratic political apparatus that distinguishes between policy development and policy implementation, with the latter remaining in the hands of elected (human) representatives (34).

In fact, our approach deliberately involves human researchers "in the loop" at multiple stages (in addition to human participants, who provide data from which the agent ultimately learns). This includes an end stage that involves interpreting the mechanism designed by the AI, and using standard metrics to study its economic properties (e.g. the extent to which it promotes productivity vs. equality). However, it also includes various stages within the design process. For example, humans are responsible for choosing the space of rival mechanisms. An election is a popularity contest among policies or representatives: in our pipeline, humans retain control in part by specifying the competitors (in the current experiment, this is the space of mechanisms that lie on the ideological manifold). In our vision of its deployment, thus, human policy makers remain the final arbiters of whether any new mechanism is likely to be safe, viable, fair or effective, and no final autonomy over governance is ceded to the AI.

A second important point concerns the interpretability of the AI-designed mechanism (9). We deliberately hampered the mechanism designer by denying it activation memory. This means that the mechanism it designed (HCRM) can be transparently described in just two dimensions (rather than, say, being a complicated nonlinear function of the choice history of different players). This is a level of complexity that is similar to the human-generated theories of distributive justice that we use as baselines. Encouraging a more interpretable mechanism has at least two advantages. Firstly, it made the agent more transparent to the human players. In fact, humans deemed the agent to be "more transparent and predictable" than the alternative AI-designed mechanism (rational mechanism), as well as (perhaps incongruously) than strict egalitarian. Secondly, the lack of memory has implications for user privacy.

Inputs to the agent were designed to be entirely “slot equivariant”, meaning that the mechanisms treated each player’s input independent of its ‘slot’ (ie. if a player is Player 1, 2, 3 or 4). The agent’s input pertained to the distribution of contributions rather than contributions from individuals themselves. Coupled with the lack of memory, this means that the agent is barred from tracking information about a particular player’s history of contributions within the game. The agent applies a general standard to everyone as redistribution is not tied to individual players' identities or history. The slot equivariance also prevents the agent to enact any policy a priori disadvantages any player position ie ‘never distribute anything to Player 2’.

Finally, questions might be raised by our emphasis on a democratic objective as a method for value alignment. Democratic AI inherits from other democratic approaches a tendency to enfranchise the majority at the expense of the minority: the “tyranny of the majority” (35). This is particularly pertinent given the pressing concern that AI might be deployed in way that exacerbate existing patterns of bias, discrimination or unfairness in society (36). In our investment game, we sampled endowment conditions to match plausible real-world income distributions, where the disadvantaged inevitably outnumber the advantaged – and so for the specific question of distributive justice that we address, this problem is less acute. However, we acknowledge that if deployed as a general method, without further innovation, there does exist the possibility that (just like in real-world democratic systems) it could be used in a way that favors the preferences of a majority over a minority group. We take this risk seriously and note that, just like in the real world, there are no straightforward solutions to this. However, we would also note (without necessarily endorsing this perspective) the tools that are used in our project at least lend themselves to the implementation of various forms of correction that might be desirable to prevent disenfranchisement of minorities. For example, it would (in theory) be possible to augment the cost function in a way that hybrid policies, for example in which a democratic objective was mixed with hard or soft constraints that encouraged fairness or protected specific groups. We are not advocating for or against such an approach, but merely pointing out that our research pipeline does not preclude it.

Supplementary Figure 1. Comparison of virtual human players and human behaviour

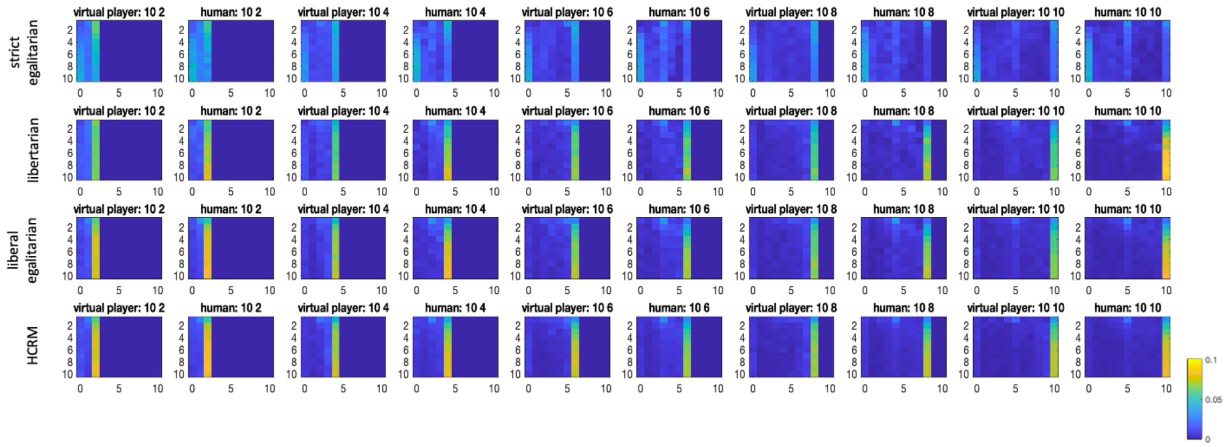

Supplementary Figure 1. Each plot shows the normalized distribution of coins contributed (x-axis of each plot, from 0-10) over trials (y-axis of each plot, from 1-10), under different endowment conditions and mechanisms (rows top to bottom: strict egalitarian, libertarian, liberal egalitarian, HCRM). Note the similarity between "virtual player" and "human" plots for the same endowment condition / mechanism.

Supplementary Figure 2. Predictors of voting

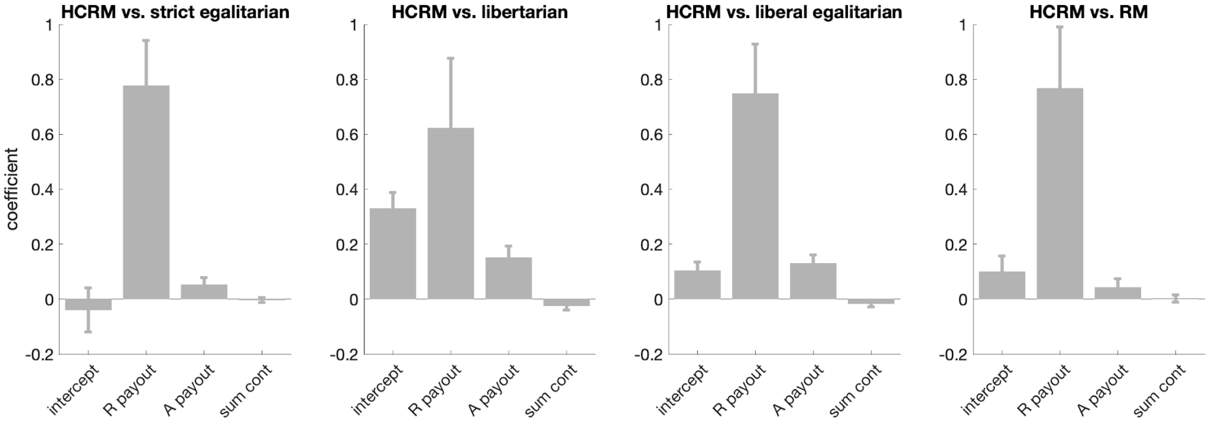

Supplementary Figure 2. Each panel shows the beta coefficients for a logistic regression predicting votes for the agent vs. a rival baseline (see panel titles) as a function of 4 competitive predictors. "R payout" is the sum of payouts relative to endowment; "A payout" is the sum of absolute payouts; and "sum cont" is total sum of contributions. In all 4 experiments, relative payout was the strongest predictor, and it was significant in all cases. Full statistics are provided in the table below. Bars are 1 S.E.M.

rival	Intercept t statistic	Intercept p-value	R payout t statistic	R payout p-value	A payout t statistic	A payout p-value	sum cont t statistic	sum cont p-value
strict egalitarian	-0.5	n.s.	4.7	< 0.001	1.93	n.s.	-0.3	n.s.
libertarian	5.57	< 0.001	2.45	< 0.02	3.72	< 0.001	-1.7	n.s.
liberal egalitarian	3.21	< 0.002	4.15	< 0.001	5.31	< 0.001	-1.5	n.s.
rational mechanism	1.81	n.s.	3.41	< 0.001	1.29	n.s.	0.16	n.s.

As can be seen, relative payout is the only variable that consistently predicts votes against all 4 baseline mechanisms. This implies that participants consistently normalized their estimates of how generous the agent was in paying out to them by the endowment they initially received. We note that against libertarian and liberal egalitarian the intercept is also reliable. This means that in these conditions, there were additional (unmodelled) variables that predict voting. We can account for some of this variance by including information from the debriefing questionnaire (see Supplementary Figure 6).

Supplementary Figure 3. Relative payouts under each mechanism.

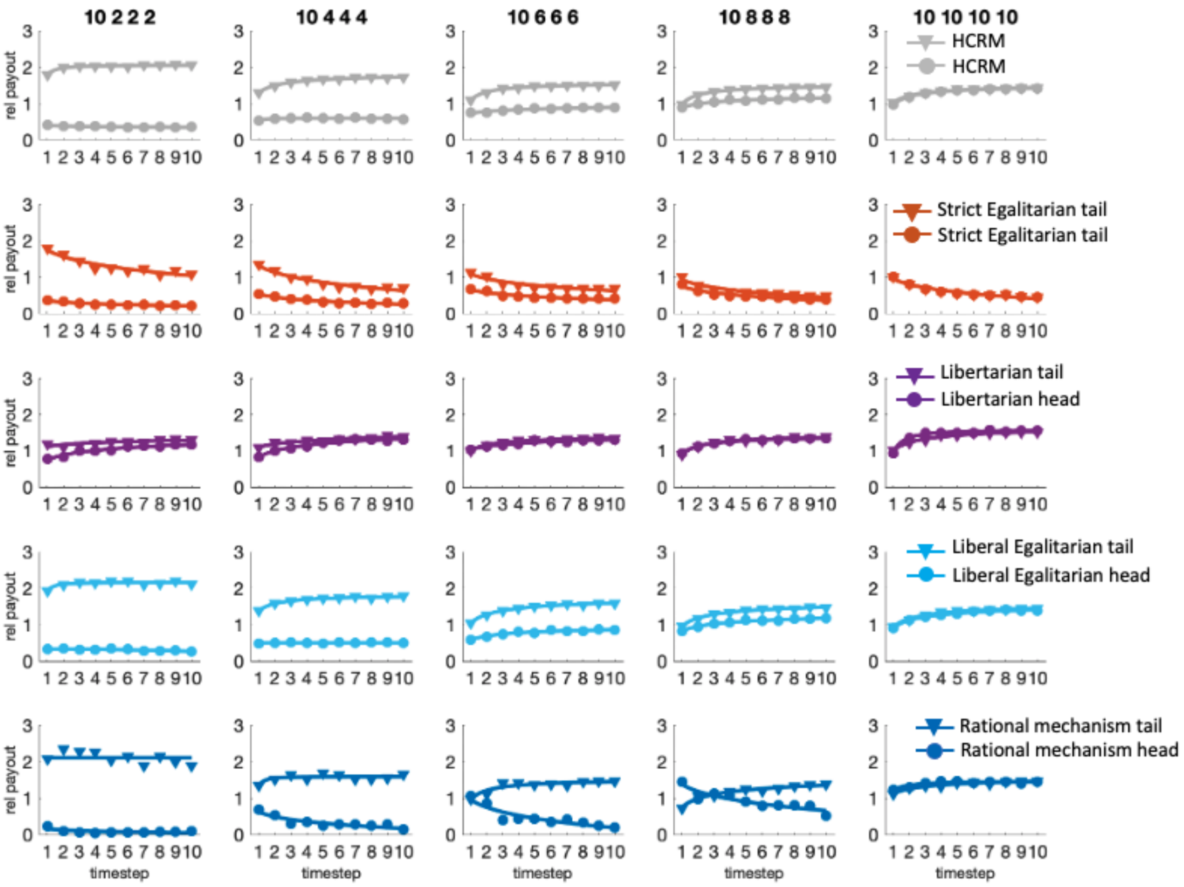

Supplementary Figure 3. Mean relative payout (y_i/e_i) to head player (circles) and tail players (triangles) over each round within the game (x-axis) for the 5 different endowment conditions (columns). Each row is a mechanism. Data are taken from blocks 2-3. The top row is the average of payouts made by the agent against all rivals. Rows 2-5 are payouts made under strict egalitarian, libertarian, liberal egalitarian, and the rational mechanism respectively.

Supplementary Figure 4. Relative contributions under each mechanism.

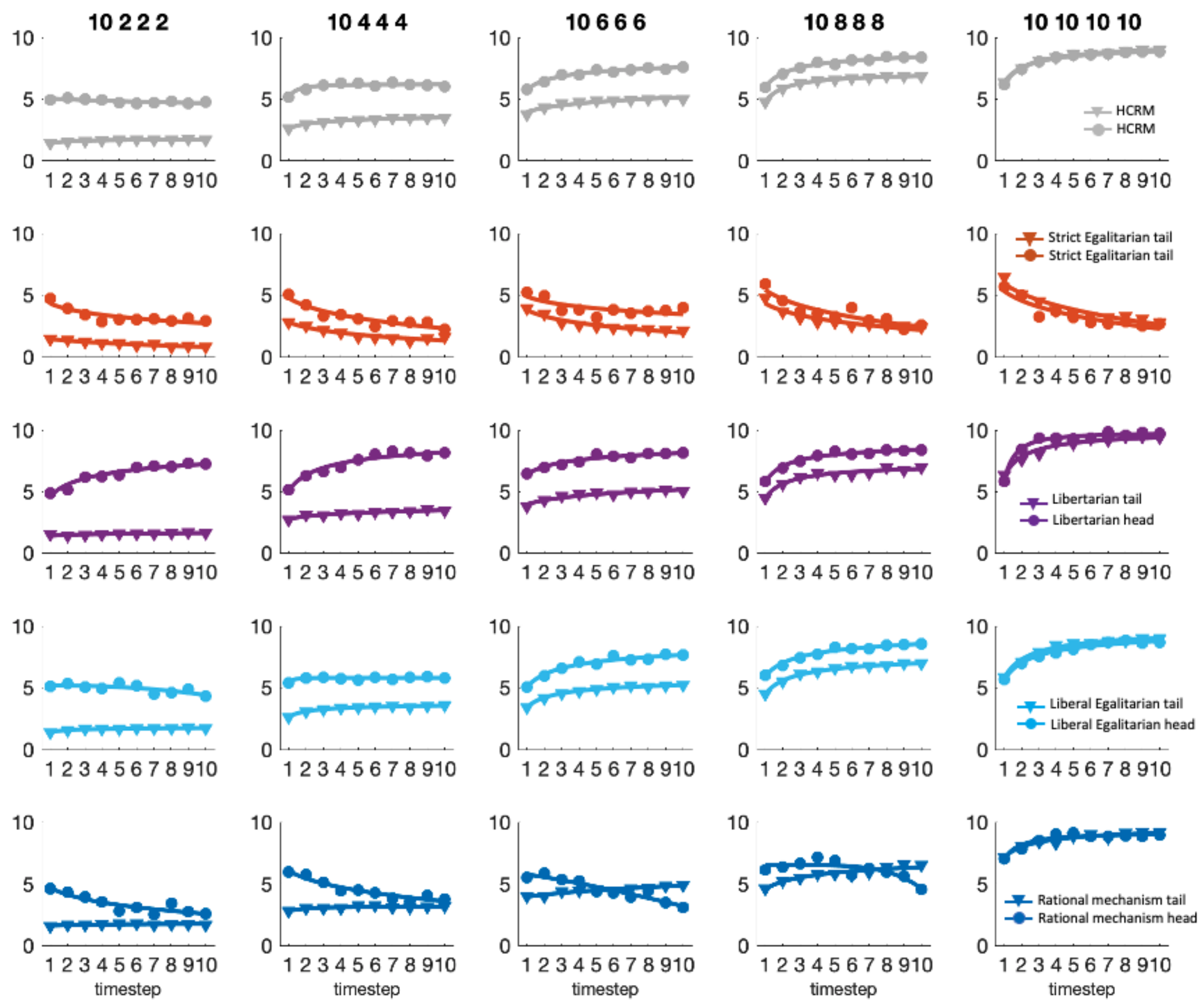

Supplementary Figure 4. Mean contributions made by head player (circles) and tail players (triangles) over each round within the game (x-axis) for the 5 different endowment conditions (columns). Each row is a mechanism. Data are taken from blocks 2-3. The top row is the average of contributions made by the agent against all rivals. Rows 2-5 are payouts made under strict egalitarian, libertarian, liberal egalitarian, and the rational mechanism respectively.

Supplementary Figure 5. Distribution of Gini Coefficients for each mechanism

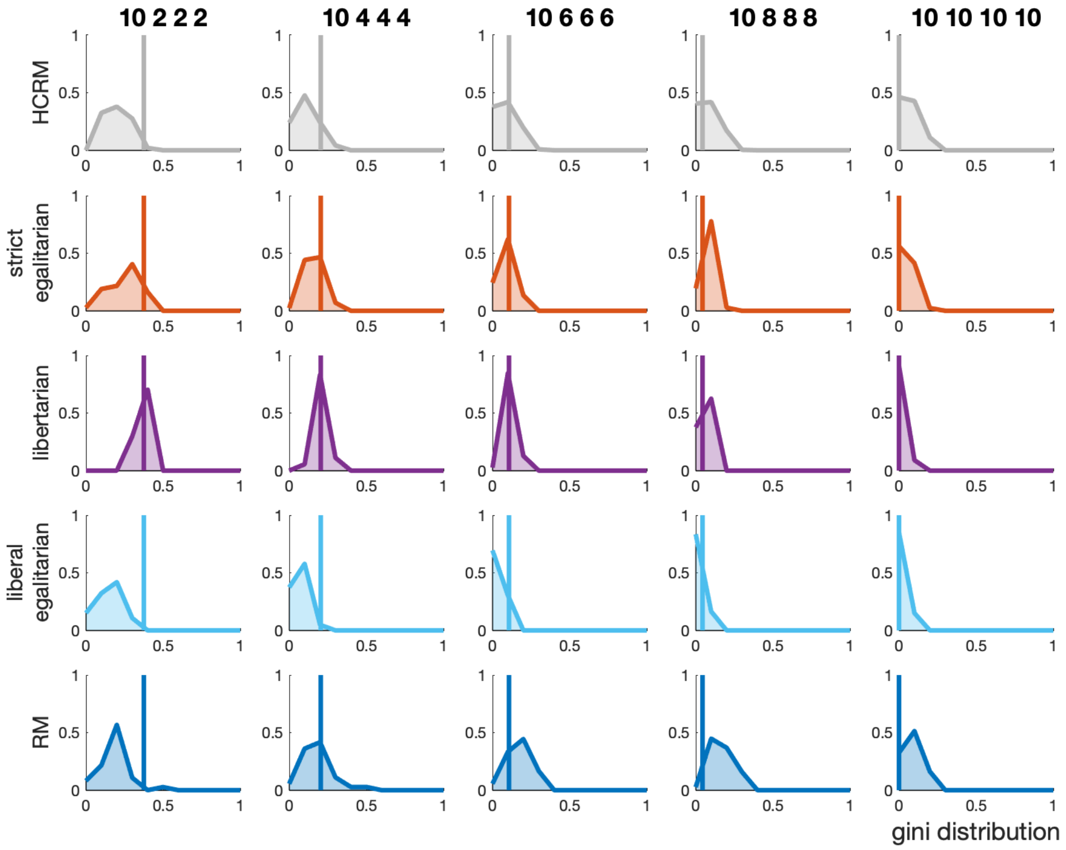

Supplementary Figure 5. Distributions of Gini coefficients computed from total return (the return to each player is their payouts plus their coins retained in the private fund) to the 4 players at the end of each game. Distributions are normalised so that they sum to one (over ten bins from 0-1). Each column is an endowment condition, and each row is a mechanism. The line shows the Gini for the initial endowment provided on each trial. Lower Gini coefficients imply greater equality.

Supplementary Figure 6. Debriefing Survey results

Debrief results. After voting, but before playing the final round, players completed a debriefing survey, which helped us understand why humans voted for HCRM. The survey consisted of 7 questions, to which they were asked to answer "A" or "B".

1. Which referee will lead to everyone doing better?
2. Which referee will lead to you individually doing better?
3. Which will lead to the majority of players doing better?
4. Which referee will be fairer?
5. Which referee will be best at fostering collaboration?
6. Which referee will be more permissive or lenient?
7. Which referee will be more transparent or predictable?

Full percentage votes for HCRM for each question (in each endowment condition and against each rival mechanism) are shown below (pink plots). However, we start by noting that positive response to all questions except q.6 predicted voting (all t-values > 4, all p-values < 0.001). Question 2 was by far the strongest predictor ($t \sim 26$, $p \sim 0$) confirming that people's voting was mostly, but not exclusively, self-interested.

In the plots below, the format is identical to Figure 2 in the main text.

'Which referee will lead to everyone doing better?'

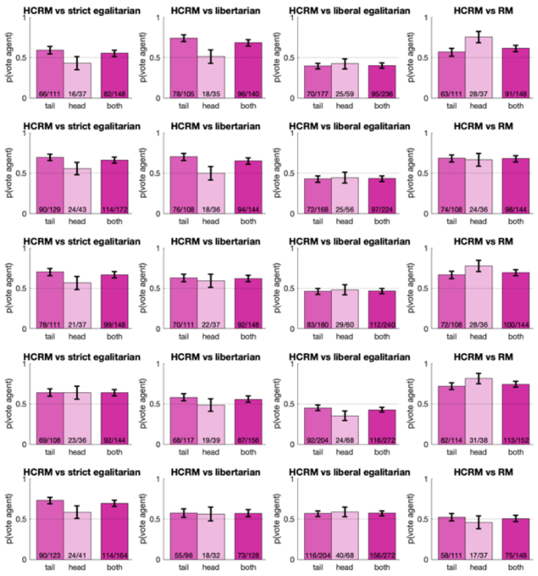

Which referee will lead to you individually doing better?

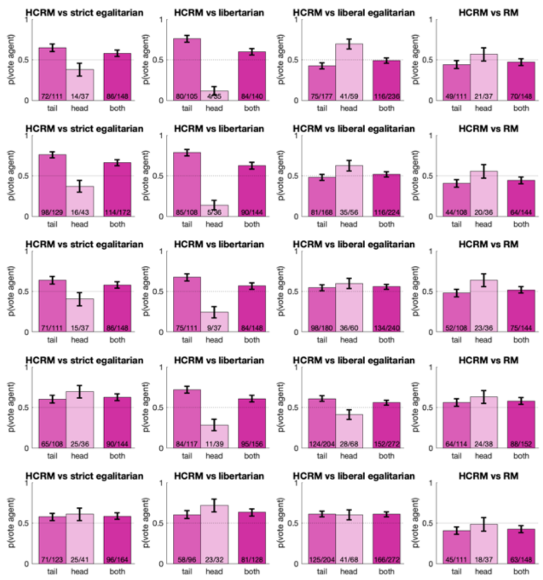

Which will lead to the majority of players doing better?

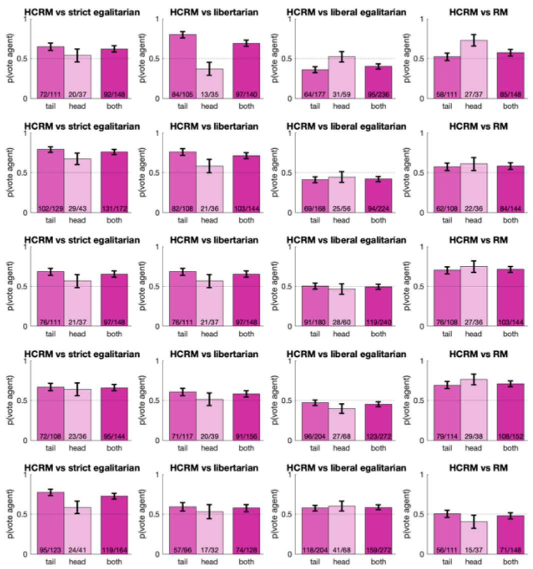

Which referee will be fairer?

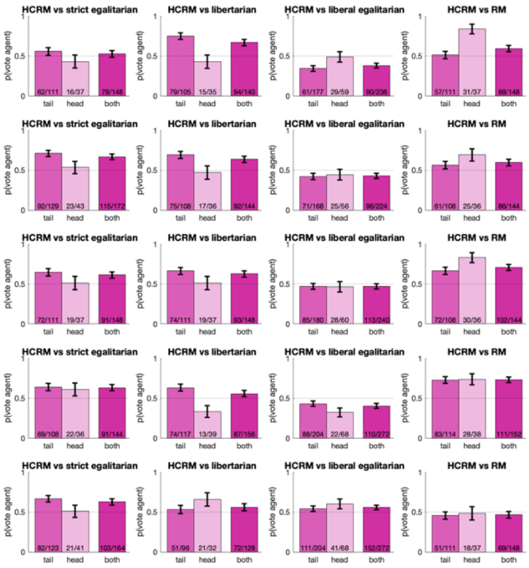

Which referee will be best at fostering collaboration?

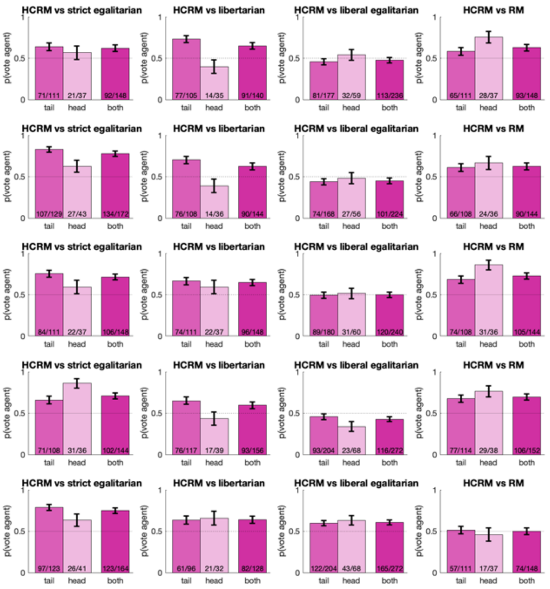

Which referee will be more permissive or lenient?

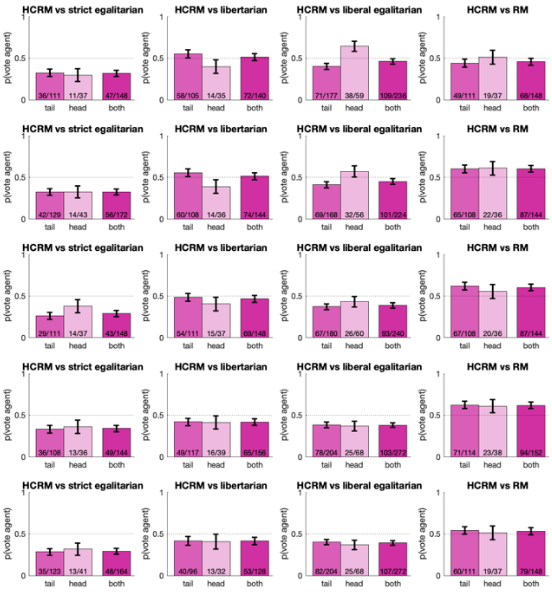

Which referee will be more transparent or predictable?

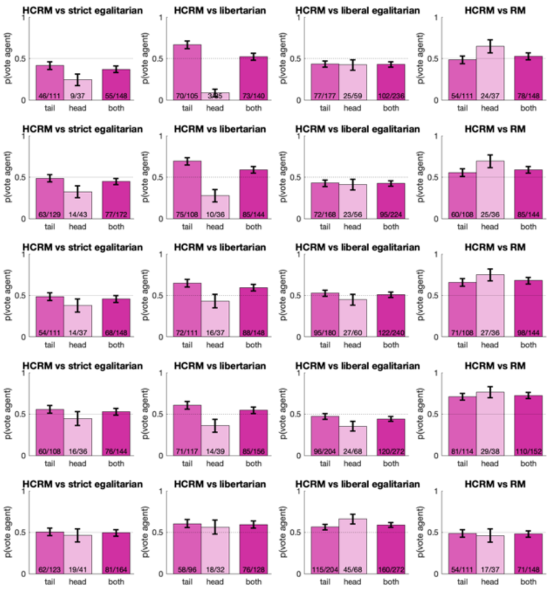

Supplementary Figure 7. Contribution distribution for each endowment and timepoint

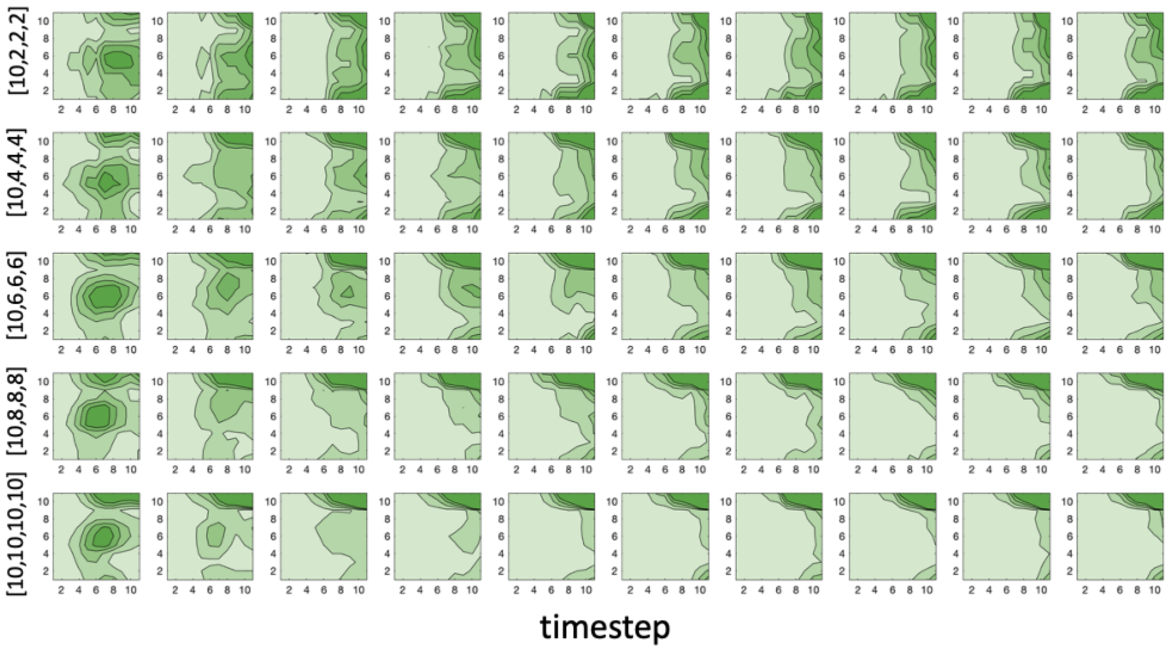

Supplementary Figure 7. Relative contribution distributions from the head player (y-axis) and tail player (x-axis), for each endowment condition (row) and round (or "timestep") within the game, averaged over all mechanisms. Darker green shading implies greater density, i.e. a higher frequency of contributions. For example, the approximately trimodal contribution from the head player on round 1 occurs because most players contribute none, half, or all their endowment (0, 5 or 10 coins) on the first round.

Supplementary Figure 8. Beach plots for return to tail / head player

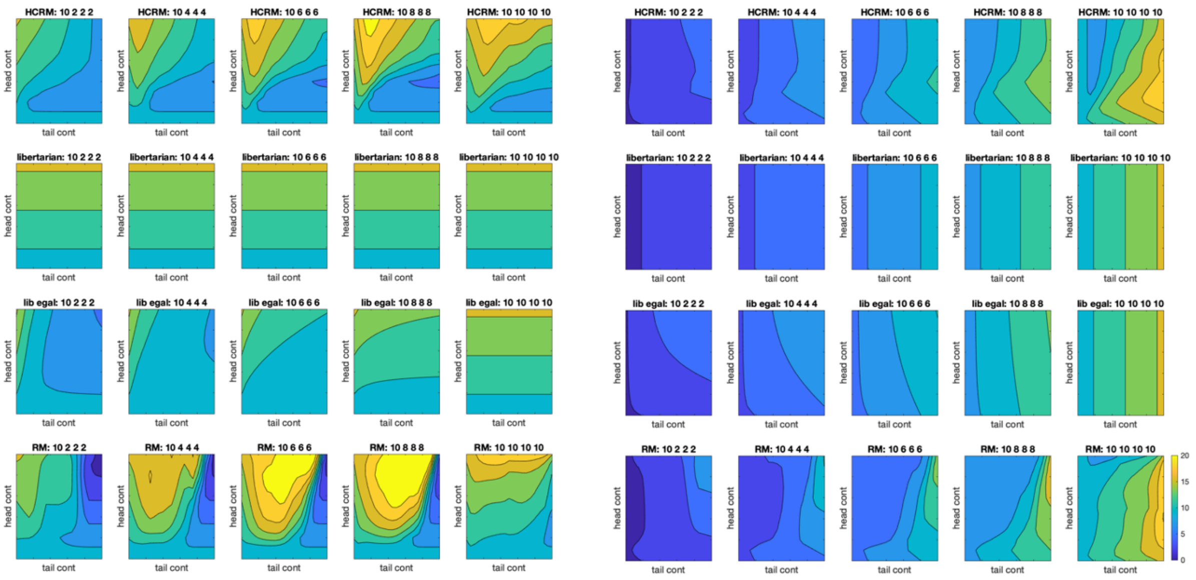

Supplementary Figure 8. Alternative beach plots describing each mechanism in terms of the return (i.e. payout plus residual endowment) for tail player (left 5 x 5 grid) and head player (right 5 x 5 grid). Units are coins (0-20). Each subplot in the 5 x 5 grid shows return to the tail (left plots) or head (right plots) players as a function of the contributions off the head player (y-axis of each subplot) and tail player (x-axis of each subplot). For example, straight lies horizontal (vertical) for libertarian policy indicate that contributions only depend on tail (head) player. Note that the HCRM creates a strong incentive for the head and tail players to compete to contribute more.

Supplementary Figure 9. The ideological manifold for each endowment condition.

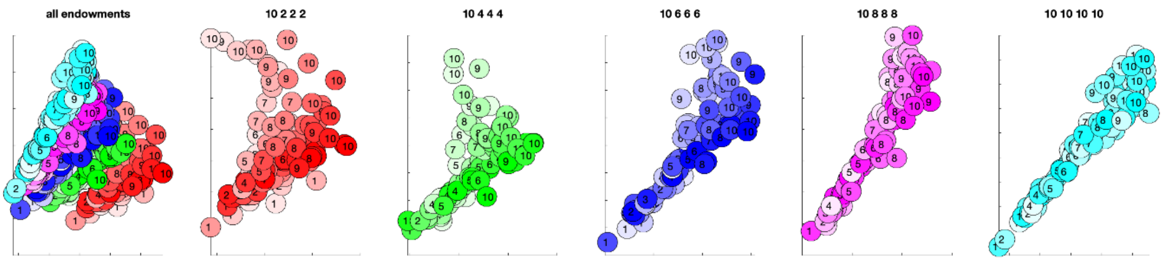

Supplementary Figure 9. The panel on the left shows the ideological manifold for all 5 endowment conditions together, using the color scheme from panels 2-6. Each panel 2-6 shows the ideological manifold for a specific endowment condition. Each dot is a mechanism. This mechanism was deployed with virtual players and we recorded and concatenated the average (relative) payout to head and tail player over 10 trials (20 features). This was used to determine the similarity among all 100 mechanisms, sampled from the 10 x 10 space defined by bins of mechanism parameters v and w. Panel 2 (second from the left) is shown in Figure 1b of the main text.

S10. Theoretical analysis of the game

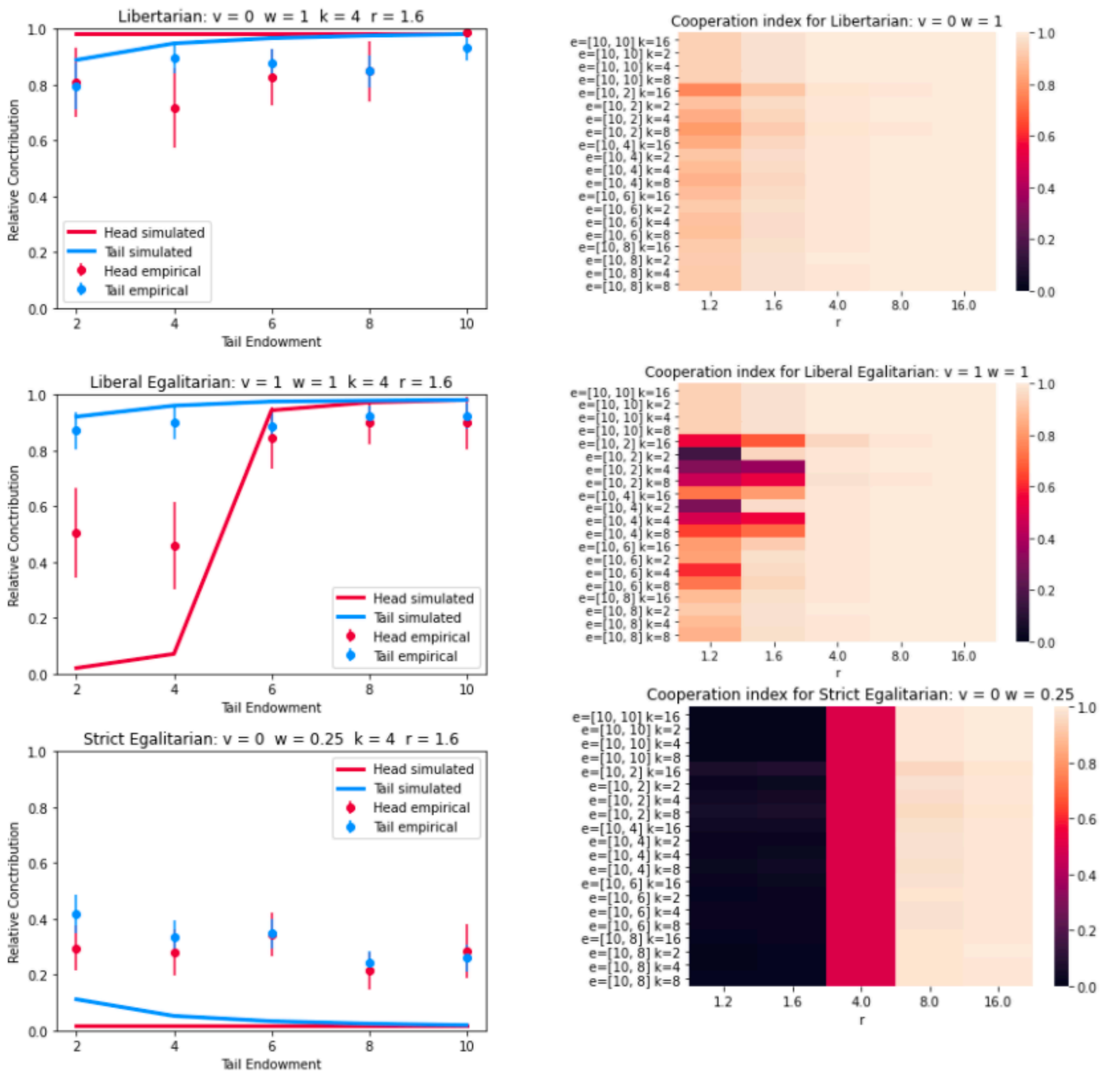

The plots explore simulation results involving the three baseline mechanisms: libertarian (top row) liberal egalitarian (middle row) and strict egalitarian (bottom row). The plots in the left column show the estimated Nash relative contribution (after convergence) for head and tail players in simulation with $r = 1.6$ and $k = 4$ (lines). This is overlaid on the true observed relative contributions from human players on the last round (i.e. timestep 10) with the corresponding mechanism. We see the results are qualitatively congruent, but overall human players' contributions exhibit a central tendency, as would be expected under noise. The right column plots a 'cooperation index' that summarizes the behaviour of both head and tail players (the fraction of available coins that were contributed by all players) for the 3 mechanisms. On the x axis the growth factor r of the pool is varied. The y axis varies settings of the number of tail players ($k = 2, 4, 8$ or 16) and the endowment given to head and tail (e.g. [10 2] is 10 coins to the head player, 2 to the tail players).

Table S1. Tables of votes.

	Votes received by HCRM (human players) against each baseline			
Endowment condition	Strict Egalitarian	Libertarian	Liberal Egalitarian	Rational Mechanism
[10 2 2 2]	84/148 (56.8%)	92/148 (62.1%)	162/372 (43.5%)	82/148 (55.4%)
[10 4 4 4]	121/172 (70.3%)	96/144 (66.6%)	172/332 (51.8%)	74/144 (51.4%)
[10 6 6 6]	93/148 (62.8%)	91/156 (58.3%)	191/328 (58.2%)	96/144 (66.7%)
[10 8 8 8]	99/144 (68.8%)	92/160 (57.5%)	186/364 (51.1%)	98/152 (64.5%)
[10 10 10 10]	116/164 (70.7%)	79/132 (59.9%)	240/372 (64.5%)	71/148 (47.9%)

Table S1a. Votes received by HCRM against each rival mechanism (real huma data). Raw numbers (x out of n votes, upper row) and percentage (lower row), for each endowment condition.

	Votes received by HCRM (human players) against each baseline		
Endowment condition	Strict Egalitarian	Libertarian	Liberal Egalitarian
[10 2 2 2]	60.5%	60.6%	51.3%
[10 4 4 4]	61.7%	54.6%	51.0%
[10 6 6 6]	64.4%	53.0%	51.6%
[10 8 8 8]	64.2%	52.3%	51.5%

[10 10 10 10]	67.5%	52.1%	51.9%

Table S1b. Percentage votes received by HCRM against each rival mechanism for the virtual players. No counts are included because virtual players can be replicated as many times as required.

Table S2. Results of the round robin tournament ("meta-game")

(v=0, w=0)	0.500	0.503	0.500	0.415	0.414	0.410	0.373	0.347	0.347
(v=½, w=0)	0.497	0.500	0.503	0.418	0.417	0.416	0.372	0.349	0.343
(v=1, w=0)	0.500	0.497	0.500	0.417	0.415	0.413	0.374	0.349	0.346
(v=0, w=½)	0.585	0.582	0.583	0.500	0.499	0.498	0.451	0.424	0.424
(v=½, w=½)	0.586	0.583	0.585	0.501	0.500	0.503	0.453	0.428	0.425
(v=1, w=½)	0.590	0.584	0.587	0.502	0.497	0.500	0.452	0.431	0.421
(v=0, w=1)	0.627	0.628	0.626	0.549	0.547	0.548	0.500	0.475	0.467
(v=½, w=1)	0.653	0.651	0.651	0.576	0.572	0.569	0.525	0.500	0.494
(v=1, w=1)	0.653	0.657	0.654	0.576	0.575	0.579	0.533	0.506	0.500

Table S2 Average number of votes given by virtual players for each mechanism generated from the linear redistribution space. We defined a set of 9 mechanisms by exhaustively combining values of v and w in {0, ½, 1}. We created a 9 x 9 payoff matrix corresponding to the average number of votes achieved by each mechanism by exhaustively combining these 9 mechanisms in pairs. These are shown here as win probabilities. The order of mechanisms is matched for rows and columns (leading to a value of 0.5 in the diagonal). The main finding of the meta-game is that liberal egalitarian ($v = 1, w = 1$) is a dominant strategy, meaning that it is the strategy achieving the highest number of votes for each player (e.g. rows) independently of the strategy of the opponent (e.g. columns).
