# Supplementary material for: Human-centred mechanism design with Democratic AI
Source: Nat Hum Behav. 2022 Jul 4;6(10):1398–407. doi: 10.1038/s41562-022-01383-x (PMC9584820; doi:10.1038/s41562-022-01383-x)
Supplement: Supplementary file 2 — Reporting Summary [file 41562_2022_1383_MOESM2_ESM.pdf]

nature portfolio

Corresponding author(s): Chris Summerfield

Last updated by author(s): April 8 2022

Reporting Summary

Nature Portfolio wishes to improve the reproducibility of the work that we publish. This form provides structure for consistency and transparency in reporting. For further information on Nature Portfolio policies, see our Editorial Policies and the Editorial Policy Checklist.

Statistics

For all statistical analyses, confirm that the following items are present in the figure legend, table legend, main text, or Methods section.

n/a	Confirmed	
☐	☒	The exact sample size (*n*) for each experimental group/condition, given as a discrete number and unit of measurement
☐	☒	A statement on whether measurements were taken from distinct samples or whether the same sample was measured repeatedly
☐	☒	The statistical test(s) used AND whether they are one- or two-sided<br>*Only common tests should be described solely by name; describe more complex techniques in the Methods section.*
☐	☒	A description of all covariates tested
☐	☒	A description of any assumptions or corrections, such as tests of normality and adjustment for multiple comparisons
☐	☒	A full description of the statistical parameters including central tendency (e.g. means) or other basic estimates (e.g. regression coefficient) AND variation (e.g. standard deviation) or associated estimates of uncertainty (e.g. confidence intervals)
☐	☒	For null hypothesis testing, the test statistic (e.g. F, t, r) with confidence intervals, effect sizes, degrees of freedom and P value noted<br>*Give P values as exact values whenever suitable.*
☒	☐	For Bayesian analysis, information on the choice of priors and Markov chain Monte Carlo settings
☒	☐	For hierarchical and complex designs, identification of the appropriate level for tests and full reporting of outcomes
☒	☐	Estimates of effect sizes (e.g. Cohen's *d*, Pearson's *r*), indicating how they were calculated

Our web collection on statistics for biologists contains articles on many of the points above.

Software and code

Policy information about availability of computer code

Data collection	Data was collected using custom code.
Data analysis	Data was analysed using custom scripts. Data of the experiment as well as code to load and analyse it will be provided at https://github.com/deepmind/hcmd_dai

For manuscripts utilizing custom algorithms or software that are central to the research but not yet described in published literature, software must be made available to editors and reviewers. We strongly encourage code deposition in a community repository (e.g. GitHub). See the Nature Portfolio guidelines for submitting code & software for further information.

Data

Policy information about availability of data

All manuscripts must include a data availability statement. This statement should provide the following information, where applicable:

- Accession codes, unique identifiers, or web links for publicly available datasets
- A description of any restrictions on data availability
- For clinical datasets or third party data, please ensure that the statement adheres to our policy

Human data will be made freely available at https://github.com/deepmind/hcmd_dai

Field-specific reporting

Please select the one below that is the best fit for your research. If you are not sure, read the appropriate sections before making your selection.

☐ Life sciences ☒ Behavioural & social sciences ☐ Ecological, evolutionary & environmental sciences

For a reference copy of the document with all sections, see nature.com/documents/nr-reporting-summary-flat.pdf

Behavioural & social sciences study design

All studies must disclose on these points even when the disclosure is negative.

Study description	The study involved a quantitative experimental design.
Research sample	The sample consisted of participants on well-known crowdsourcing platforms and no demographic data was recorded. Estimates of sample sizes were created by simulations using neural networks.
Sampling strategy	The sampling strategy was convenience sampling from the crowdsourcing platforms. Sample sizes were determined by simulations based on neural networks.
Data collection	Participants performed the task computerized over the internet. The researcher was not blind to the experimental conditions but did not interact with the participants prior to them doing the task.
Timing	The data was collected between the 7th of November 2019 to the 13th of August 2021.
Data exclusions	No participants were excluded from the data.
Non-participation	Of the 4804 participants in the main experiment, 48 did not submit a vote (for unknown reasons). These were excluded from the analysis. An unknown number of participants voluntarily aborted participation in the task and were excluded before all analyses.
Randomization	Allocation was fully random.

Reporting for specific materials, systems and methods

We require information from authors about some types of materials, experimental systems and methods used in many studies. Here, indicate whether each material, system or method listed is relevant to your study. If you are not sure if a list item applies to your research, read the appropriate section before selecting a response.

Materials & experimental systems

n/a	Involved in the study	
☒	☐	Antibodies
☒	☐	Eukaryotic cell lines
☒	☐	Palaeontology and archaeology
☒	☐	Animals and other organisms
☐	☒	Human research participants
☒	☐	Clinical data
☒	☐	Dual use research of concern

Methods

n/a	Involved in the study	
☒	☐	ChIP-seq
☒	☐	Flow cytometry
☒	☐	MRI-based neuroimaging

Human research participants

Policy information about studies involving human research participants

Population characteristics	We did not collect demographic information of participants.
Recruitment	Participants were self-selected by being users of popular crowdsourcing platforms in the UK and USA. The nature of the sample, and its effect on our behavioral results are unknown. However, our experiment makes no claims about a specific effect in relation to any population, but rather demonstrated a modelling pipeline that can effectively capture the behavior of an arbitrary subpopulation.
Ethics oversight	The study was approved by HuBREC (Human Behavioral Research Ethics Committee), which is a research ethics committee run within Deepmind but staffed/chaired by academics from outside the company.

Note that full information on the approval of the study protocol must also be provided in the manuscript.
